# A Systematic Review of Studies Measuring and Reporting Hearing Aid Usage in Older Adults since 1999: A Descriptive Summary of Measurement Tools

**DOI:** 10.1371/journal.pone.0031831

**Published:** 2012-03-27

**Authors:** Elvira Perez, Barrie A. Edmonds

**Affiliations:** 1 National Biomedical Research Unit in Hearing, National Institute for Health Research (NIHR), Nottingham, United Kingdom; 2 School of Clinical Sciences, University of Nottingham, Nottingham, United Kingdom; Stanford University School of Medicine, United States of America

## Abstract

**Objective:**

A systematic review was conducted to identify and quality assess how studies published since 1999 have measured and reported the usage of hearing aids in older adults. The relationship between usage and other dimensions of hearing aid outcome, age and hearing loss are summarised.

**Data sources:**

Articles were identified through systematic searches in PubMed/MEDLINE, The University of Nottingham Online Catalogue, Web of Science and through reference checking. Study eligibility criteria: (1) participants aged fifty years or over with sensori-neural hearing loss, (2) provision of an air conduction hearing aid, (3) inclusion of hearing aid usage measure(s) and (4) published between 1999 and 2011.

**Results:**

Of the initial 1933 papers obtained from the searches, a total of 64 were found eligible for review and were quality assessed on six dimensions: study design, choice of outcome instruments, level of reporting (usage, age, and audiometry) and cross validation of usage measures. Five papers were rated as being of high quality (scoring 10–12), 35 papers were rated as being of moderate quality (scoring 7–9), 22 as low quality (scoring 4–6) and two as very low quality (scoring 0–2). Fifteen different methods were identified for assessing the usage of hearing aids.

**Conclusions:**

Generally, the usage data reviewed was not well specified. There was a lack of consistency and robustness in the way that usage of hearing aids was assessed and categorised. There is a need for more standardised level of reporting of hearing aid usage data to further understand the relationship between usage and hearing aid outcomes.

## Introduction

In the UK, it is estimated that 41.7% of adults aged 50 years or over has some form of hearing loss [1]. Presbycusis (age-related sensorineural hearing loss) is the most common type of hearing loss and it affects 25–43% of people aged between 65 and 74 years and 40–63% of those aged over 75 years [2], [3]. The most common form of treatment for hearing loss in adults is the provision of a hearing aid. It is estimated that about six million people in the UK could benefit from using a hearing aid, but only 1.4 million people in the UK who currently own hearing aids wear them regularly [4]. Hearing loss increases the need for more formal support (e.g., meals on wheels) in the elderly and it impacts negatively on independence by increasing reliance on community/family support [5]. Hearing loss also has an impact on communication, social interaction and self-sufficiency [6]. If left untreated (either through non-detection of the problem or non-adherence with the treatment) hearing loss can further precipitate social, physical and psychological decline [7] and will likely result in wasted resources (i.e., clinical time and un-used hearing aids). Consequently, when hearing aids are prescribed for the treatment of hearing loss it is important to ensure that patients are provided with regular follow up appointments to monitor their success [8].

The success of hearing aid provision as a treatment for hearing loss, like any health-care intervention, largely depends on two factors. First, the intervention should be capable of providing the patient with a favourable change in their condition. Second, the patient must comply with the intervention programme in order to have a chance of receiving that benefit. Indeed, there are a number of tools available for monitoring patient outcomes following provision of a hearing aid which seek to determine how often a hearing aid is used, whether the patient has experienced any changes in speech understanding, or other changes to quality of life. The amount of time spent wearing a hearing aid has been reported to be positively associated with the benefit that the hearing aid provides (i.e., an improvement in speech understanding) and levels of patient satisfaction [9], [10]. However, Humes et al [11] performed a principal component analysis of seven dimensions of hearing aid outcome (i.e., subjective benefit, aided performance, usage, objective benefit, speech in noise, handicap benefit and judgements of sound quality) which indicated that hearing aid usage is a distinct and relatively independent dimension of hearing aid outcome.

Bentler and Kramer [12] in an overview of audiological-focused self-report outcome measures identified three self-report inventories, out of 33, that assessed hearing aid usage: the Hearing Problem Inventory [13], the Hearing Aid User Questionnaire (HAUQ) [14], and the Glasgow Hearing Aid Benefit Profile (GHABP) [15]. In the last decade, however, the number of outcome instruments has increased considerably. Moreover, there has been a step change in hearing aid technology available in this period which has enabled more sophisticated devices (e.g., the advent of hearing aids capable of digital signal processing) to enter the market. Furthermore, audiology departments in the UK adopted a modernised hearing service which has allowed for greater levels of patient-clinician interaction, provision of digital hearing aids and the option of bilateral fittings. However, there are currently no recognised guidelines in the UK for managing the rehabilitation of patients with hearing loss using hearing aids nor is there a gold standard outcome measure for evaluating the success of auditory rehabilitation. Consequently, one of the dilemmas faced by clinicians and researchers is how to assess hearing aid usage and which methods to use. Self-reports measures are criticised because they are subject to socially desirable responses and may provide inaccurate responses. However, it has also been argued that objective alternatives (i.e., hearing aid data logs and battery consumption) are not free from error either, as users can forget to turn off the devices [16].

In this manuscript, we provide a descriptive overview of studies that were published since 1999 pertaining to usage of hearing aids in older listeners with sensori-neural hearing loss. We examined how usage has been measured and reported and how usage of hearing aids related to other dimensions of hearing aid outcomes. Information about study design, outcome instruments, measures of usage, and cross validation of data was extracted from research manuscripts identified using a systematic search. The purpose of this systematic review was consolidate this information into one source, to grade the quality of the evidence reported, and to identify trends in the data reported that might inform clinical guidelines or the direction of future research. We hope that clinicians and researches will find the review interesting and of help when deciding on how they should measure and report hearing aid usage in future reports.

## Methods

The Preferred Reporting Items for Systematic reviews and Meta-Analyses (PRISMA) statement checklist [17] was used to improve the reporting of this systematic review as suggested by the NHS Centre for Reviews and Disseminations [18].

### Search strategy

A systematic search strategy, following the principles published by the NHS Centre for Reviews and Dissemination [18] was used to identify potential articles. The University of Nottingham Online Catalogue, PubMed/MEDLINE, and Web of Science (including Science Citation Index Expanded, Social Sciences Citation Index, Arts & Humanities Citation Index, and Conference Proceedings Citation Index- Science) electronic databases were searched for peer-reviewed articles published between 1999–2011 using the search term “hearing aid” in conjunction with each of the following terms: “outcome”, “use”, “usage”, “non-use”, “compliance”, “satisfaction” and “benefit”. Database searches were conducted in April 2010 and updated in September 2011. Further ‘hand searches’ of the key subject journals (to identify articles published since August 2011 that may not yet have been registered on a database) were conducted in October 2011.

### Inclusion criteria and study selection

The objective of this review was to examine the reporting of hearing aid usage as an outcome measure and the factors that contribute to hearing aid usage. The author's current research interests lie with the auditory rehabilitation of presbycusic patients. In order to align the scope of this review with these research interests, we restricted eligibility of studies for review using a set of pre-specified inclusion criteria. Studies had to include participants aged fifty years or over with sensori-neural hearing loss, that had been provided with an air conduction hearing aid for the treatment of their hearing loss. Although no limitations were placed on the nature of the primary outcome measure reported, studies were only included if a measure of hearing aid usage was recorded. In addition, no constraints were placed on acceptable study design. While randomised control trials would be preferred as they give the highest level of evidence, it was anticipated that not many studies would meet this criteria.

Titles and abstracts of articles identified by the search strategy were screened to determine whether the study matched the inclusion criteria. Where evidence for inclusion of a manuscript was in doubt from the title and abstract, the full text was screened. The reference lists of all manuscripts matching the inclusion criteria were searched to find additional manuscripts for inclusion in the review.

### Data extraction

Data extraction was performed independently by both authors using an electronic reporting form developed specifically for this purpose; any differences in reporting were reconciled by jointly revisiting each publication. The reporting form was used to capture the following information: aims, study design, sample size, sampling issues, audiometric data and participant's age at time of outcome, timing of post-fitting follow-ups, outcome instruments, usage assessments, usage data and associations with other variables.

### Quality assessment criteria

All studies that met the inclusion criteria were subjected to a quality assessment exercise. Our approach to quality assessment was informed by NHS guidelines for systematic reviews [18]. The quality of each study was determined against six pre-specified criteria: i) study design, ii) use of appropriate outcome measures (i.e. standardised questionnaires and tests), iii) reporting of usage data, iv) cross validation of usage estimates (i.e. were estimates of usage obtained using multiple alternative methods or repeated measures over a number of visits), v) reporting of participant age and vi) reporting of audiometric data. While the first criterion assesses the overall methodological quality of the studies reviewed, the other criteria influence the reader's confidence in the reported usage effects in older adults with hearing loss.

Each study was given an overall grading of high, moderate, low or very low which reflected our confidence in the reported effect [19]. The grade awarded to each study was based on a number of points accrued on the six quality criteria: *High* (10–12 points), *Moderate* (7–9 points), and Low (4–6 points) and Very low (0–3 points). For each criterion, studies were awarded *0* points if the information regarding the criterion was absent or flawed on a number of levels, *1* point was awarded if the study partially met the criterion or the implementation was partially flawed, and *2* points were awarded if the study fulfilled the criterion to a high standard. Studies were independently scored by both authors and a final score was agreed after discussion. Specific examples of how points were awarded to studies for each of the six criteria are given in the relevant portions of the Results section.

It should be noted that each criterion is given equal weighting in the overall grade. That is, we consider each of the criteria as important as the next for the purpose of addressing the aims of this review. As such, studies that were graded as ‘Low’ or ‘Very Low’ quality use a combination of methods or reporting techniques which on-the-whole reduced our confidence in the results reported or limited the degree to which the results could be interpreted in the context of this review. Studies that were graded as being of ‘High Quality’ on-the-other-hand incorporated methods or levels of reporting which on-the-whole strengthened our confidence in the usage data reported and its relevance to the topic of hearing aid usage in older adults while. If a reader is interested in only a subset of the quality criteria used here, then the overall grading could be ignored entirely or replaced with a grading system that gives greater weight to one or more of the quality criteria.

## Results

### Search results

**Figure 1:**
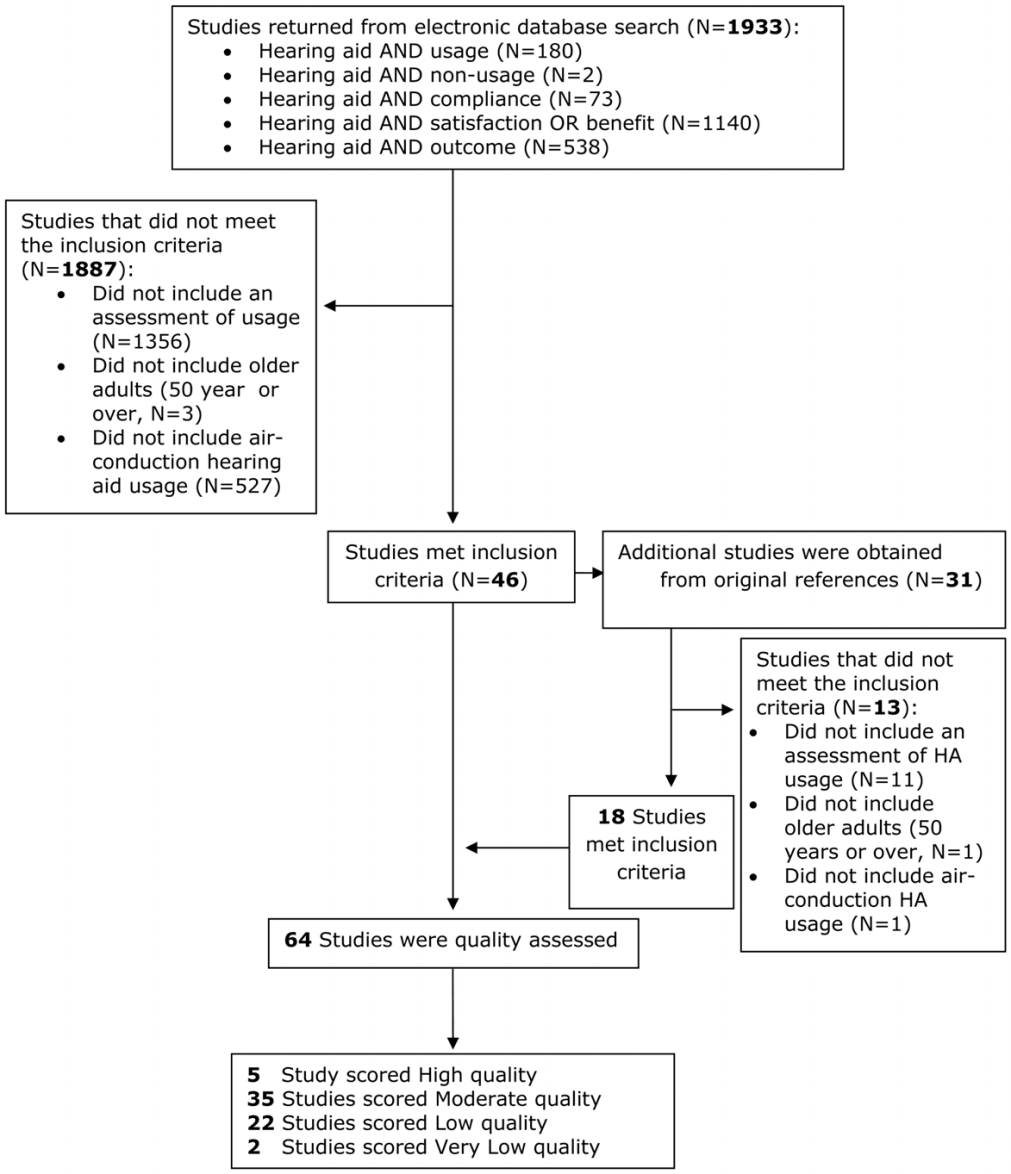
Flow diagram to illustrate the systematic review process undertaken.

The systematic search of electronic databases produced 1933 items of which 46 met the inclusion criteria for this review (see Figure 1). A further 18 eligible papers were obtained by searching the references of the original 46 giving a total of 64 research papers. The selected studies originated from 16 countries: Australia (n = 10), Canada (n = 1), China (n = 1), Denmark (n = 3), Finland (n = 4), Germany (n = 2), Hong Kong (n = 1), New Zealand (n = 2), Nigeria (n = 1), Norway (n = 1), Taiwan (n = 1), The Netherlands (n = 1), UK (n = 6), USA (n = 27), Sweden (n = 2), and Switzerland (n = 1). The information obtained at the data extraction stage is summarised in Table S1.

### Quality assessment

#### Study design

In clinical or therapeutic settings, randomisation is the ‘gold standard’ design [20] and systematic reviews often only include randomized trials [21]. When participants are not randomly allocated to groups, the detection of sampling bias is crucial for assessment of the quality of study design. Our review identified 54 observational studies of which eight were cross-sectional studies, eight randomised control trials and one crossover clinical trial. Thirty-eight studies did not specify whether any steps were taken to adjust for sampling bias. Twenty studies used only a single clinic and six studies only recruited male veterans. Other studies, however, included as many as twelve private clinics and seven public hospitals to recruit participants [22] or randomly selected participants from the public of a specific geographical area [23]. Other methods employed in order to minimise sampling bias included: contacting non-respondents, analysing potential differences between the results of different hearing clinics and surveys, randomly selected the hearing clinics involved and recruiting participants via a range of media (i.e., newspapers, advertisements, flyers, printed announcements in church bulletins, or word of mouth).

When grading the quality of evidence for study design, a score of 2 was awarded to randomised control trials (see Table S2). A score of 1 was awarded to all other study designs. A score of 0 was reserved for studies with serious methodological and design issues. In consideration of the evidence outlined above, eleven studies employed a high quality study design (scored *2* points) which utilised randomisation, control groups were a population-based study or other measures to minimise bias. A total of 30 studies were rated as medium quality as they implemented a non-optimal design but took steps to identify and address possible sources of bias. A score of 0 was awarded to 24 studies because they used a severely flawed design that failed to provide any adjustments for sample bias or randomisation of conditions.

#### Outcome instruments

**Figure 2:**
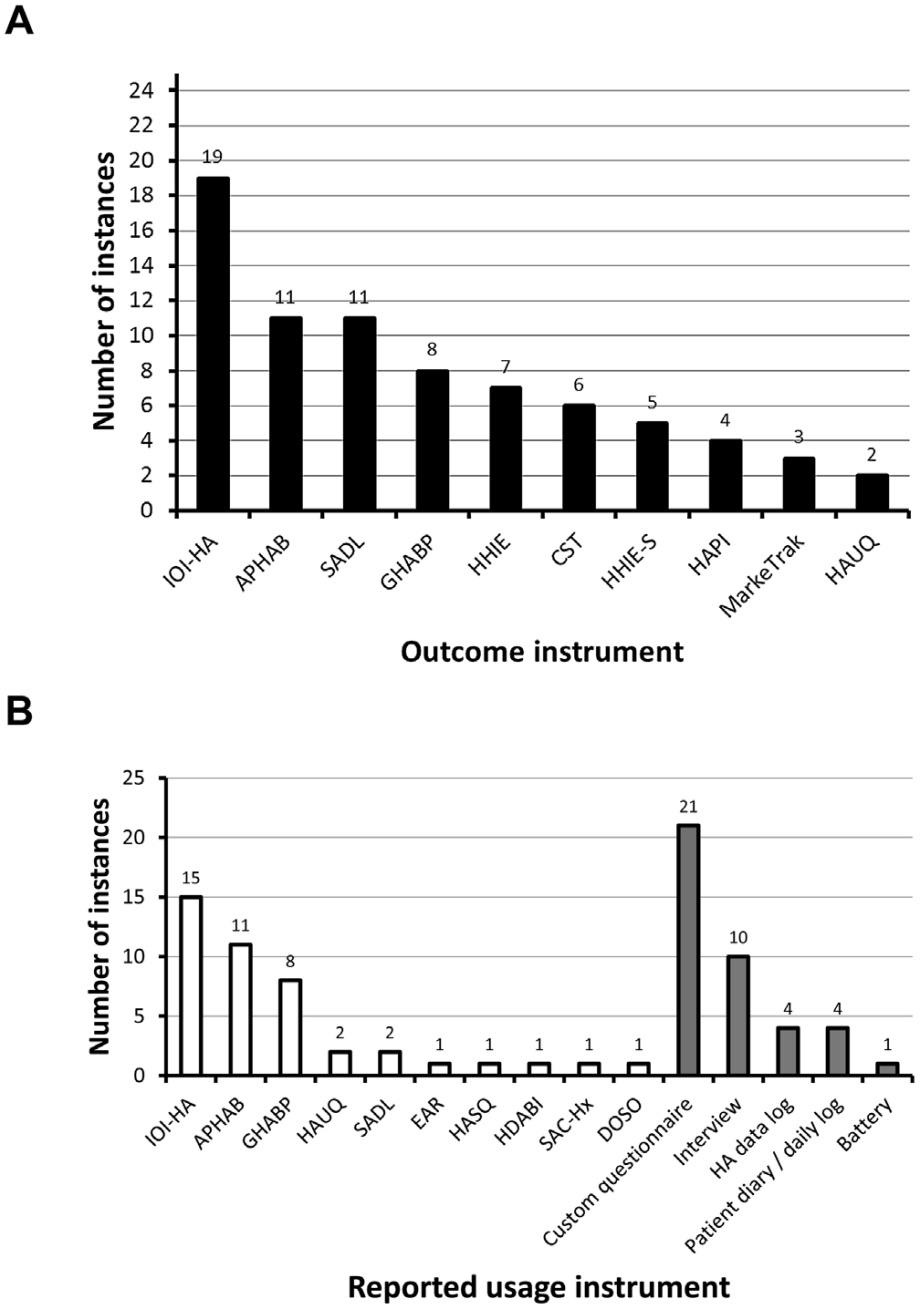
Reported instances of outcome and usage instruments. Panel A shows the top ten most used outcomes measures. Panel B shows the range of methods employed for assessing hearing aid usage. White bars indicate standardised questionnaires; grey bars indicate non-standardised methods. Abbreviations: **IOI-HA** International Outcome Inventory - Hearing Aids [32], **APHAB** Abbreviated Profile of Haring Aid Benefit [57], **SADL** Satisfaction with Amplification in Daily Life [34], **GHABP** Glasgow Hearing Aid Benefit Profile [15], **HHIE** Hearing Handicap Inventory for the Elderly [59], **CST** Connected Speech Test [36], [56], **HHIE-S** screening version [62], **HAPI** Hearing Aid Performance Inventory [54], **MarkeTrak** [63], [64] and **HAUQ** Hearing Aid Users Questionnaire [55], **EAR** Effectiveness of Auditory Rehabilitation [60], **HASQ** Hearing Aid Status Questionnaire [58], **HDABI** Hearing Disability and Aided Benefit Interview [15], **SAC-Hx** Self-Assessment of Communication [40] and **DOSO** Device Oriented Subjective Outcome Scale [61].

A large number of validated and non-validated tests and techniques were used to evaluate dimensions of hearing aid outcome (see Figure 2a for the top ten most used outcome instruments). The majority of the studies reviewed focused on dimensions of satisfaction, benefit or residual handicap as the primary outcome measure for their research question rather than hearing aid usage. Hearing aid usage was the primary outcome measure in 10 studies [16], [23], [24], [25], [26], [27], [28], [29], [30], [31]. Of the top six outcome instruments, the Glasgow Hearing Aid Benefit Profile (GHABP, [15]) and International Outcome Instrument - Hearing Aids (IOI-HA, [32]) are multi-dimensional instruments, the Abbreviate Profile for Hearing Aid Benefit (APHAB, [33]) focuses on subjective benefit, the Satisfaction and Amplification in Daily Life (SADL, [34]) focuses on satisfaction, the Hearing Handicap Inventory for the Elderly (HHIE, [35]) focuses on handicap, and the speech reception tests like the Connected Speech Test (CST, [36]) or City University of New York Nonsense Syllable Test (CUNY NST, [37]) assesses benefit objectively in the form of percentage correct, other test like the QuickSIN [38] provide a threshold for speech intelligibility (typically signal-to-noise ratio in decibels for identifying 50% of items correctly).

In grading the quality of studies based on the choice of outcome measures, we looked for evidence of multiple standardised or validated tests of outcome in our quality assessment. A total of 32 studies included multiple standardised outcome measures and were rated as being of high quality. A score of *1* was awarded to 25 studies that employed a range of non-standardised tests or only a single standardised test to determine hearing aid outcomes. Seven studies scored *0* points as hearing aid outcome was determined purely on the basis of a single non-standardised outcome measure.

#### Assessment of hearing aid usage

Hearing aid usage was assessed with standardised questionnaires in 32 of the studies reviewed, 21 studies included custom questionnaires; interview was the third most popular method for assessing usage, diaries and hearing aid data logs the fourth, while battery consumption was used in only one of the studies reviewed (see figure 2b). Data logs likely reflect the relatively recent addition of such functionality in hearing instruments. We would expect to see greater use of this digital logging feature in future studies.

The most popular standardised questionnaires for measuring usage (see Figure 2b) were the IOI-HA [32], the APHAB [33] and the GHABP [15].

### *Reporting hearing aid usage*

Both IOI-HA and GHABP categorise usage on a 5-point scale, but differ in their approach. The GHABP asks the patient to reflect on what *proportion of time for a given activity* that they use their hearing aid (acceptable response are: all of the time, about ¾ of the time, about ½ of the time, about *a ¼ of th*e time, never/not at all). The IOI-HA assesses the *average amount of time* spent wearing a hearing aid (i.e. ‘on an average day, how many hours did you use the device? Acceptable responses are: >8 hr/day, 4–8 hr/day, 1–4 hr/day, <1 hr/day, none). The distinction is subtle but potentially very important. The GHABP focuses very much on the situations important to the patient, but gives data that is difficult to compare against other studies and measures of usage (e.g., what does wearing a hearing for ¾ of the time spent watching television really mean?). The IOI-HA is perhaps less patient-needs focused, but gives data that is clearly comparable with other studies (8 hours is always 8 hours). The APHAB is a 24-item self-assessment questionnaire that reports patient's communication difficulties. It produced scores for 4 subscales; easy of communication, reverberation, background noise and aversiveness. APHAB includes a daily hearing aid section in which participants have to circle the most appropriate answer (less than 1 hour per day, 1 to 4 hours per day, 4 to 8 hours per day or 8 to 16 hours per day).

If one compares the data presented in Figure 2a with that of 2b a number of discrepancies can be observed in the number instances an outcome instrument is reported. For instance, the SADL which consists of 15 items on hearing aid performance and user satisfaction, and four additional questions concerning hearing aid experience and use, featured in a total of 11 of the studies reviewed. However, only two of these studies reported the amount of time spent wearing a hearing aid in the format dictated by the SADL: the other nine studies either reported usage in the format of another outcome instrument or failed to report usage in any form.

Custom questionnaires and structured interviews included open questions (e.g., ‘How many hours a day do you use your hearing aid?) or were built around specific duration-based categories. The format of patient diaries was not well described nor was the method used to interpret entries, but they were usually complemented by estimates of usage obtained from questionnaires like the GHABP [15], digital data logs or interviews [24].

Data regarding the usage of hearing aids was graded by employing an approach which awarded points for clear and unambiguous presentation of data. A total of 38studies recorded usage as duration in hours per day or categorized usage with a numerical value and scored *2* points for this criterion. A score of *1* was awarded to 16 studies because they categorized usage using only descriptive labels (e.g., occasionally or all of the time). Ten studies scored *0* points because they categorized usage with binary labels or did not report the amount of time participants used their hearing aids despite having collected the data (i.e., the data and format of that data was not reported in the manuscript).

#### Cross-validation of usage data

In order to further strengthen our confidence in the usage data reported, we asked whether or not efforts had been made to check the reliability/validity of the data collected. Seven studies scored *2* points for the cross-validation of usage data by combining subjective estimates (e.g., self-report diary) and objective measures (i.e., device memory or battery usage) or by assessing patients on multiple visits. Thirteen studies scored *1* point for cross validation as they compared data from two different self-report questionnaires. The remaining 44 studies did not provide any cross-validation of hearing aid usage data and scored *0* points for this criterion.

#### Reporting of participant age

In order to appreciate the relevance of each study for understanding auditory habilitation in older adults, we examined the way in which the age of participants was reported. Our strategy was to award points to studies for clear and unambiguous reporting of the data. A total of 48 studies scored *2* points for reporting data pertaining to the age of participants to a high standard (i.e., they reported the mean and standard deviation or analysed participants' data by age group). A total of 14 studies provided some details about the age of participants (e.g., minimum, maximum or range of ages) but no measures of the variance (scored *1* point). Age was not reported in two studies [39], [40]] that included war veterans (scoring *0* points).

#### Reporting of audiometric data

Audiometric data derived from pure tone hearing thresholds are necessary to establish the degree of hearing loss, and further explore how this variable might relate to usage. In order to fully understand the relevance of each study to older adults with hearing loss we examined the way in which audiometric thresholds were reported. Again, we rated the quality of studies on this criterion based on the level of reporting. Twenty-six studies scored *2* points for reporting audiometric data to a high standard including means and standard deviations per frequency band. Twenty-four provided some audiometric aspects like pure-tone average (scoring *1* point). Audiometric data was not reported in fourteen studies (scoring *0* points).

### Factors associated with the usage of hearing aids

Previous research ([41], not reviewed) showed a significant positive correlation between hearing aid use and increasing hearing loss in the elderly. However, our review indicates that this result is not reflected in the majority of studies. Thirteen studies assessed the impact of hearing loss at fitting on usage of hearing aids, but hearing loss was found to be significantly related to usage in only three of these studies [28], [42], [31].

The relationship between age and hearing aid usage was assessed in only eight studies. Two studies [28] and [43] showed a decrease in hearing aid usage with increasing age (>75 years) due to low benefit expectations, a reduction in dexterity to handle the hearing aid, or acquirement of alternative coping mechanisms (e.g., turning the volume up on the television). Five studies did not find any association between age and hearing aid usage and only one [31] found age as a significant predictor of hearing aid usage where older adults (80+) had the highest incidence of hearing aid use (25%). Complementarily, Vuorialho et al. [44] reported that the proportion of individuals with hearing aids was greater in the retired population than in the general population.

A number of the studies reviewed ([45], [46], [47]) suggested that usage was not strongly correlated with improvements in technical aspects of hearing aids made over the last decade or so. Rather, Bertoli et al. [28] suggested that difficulty in handling aids is one of the most important factors contributing to non-regular use of hearing aids. Indeed, a number of studies reviewed here ([48], [22], [45], [49]) showed that the efforts of manufacturers towards making hearing aids smaller, easier to handle and aesthetically more attractive can reduce the risk of non-regular use.

Lupsako et al. [23] noted that in countries where hearing aid provision is not state funded, hearing aid possession was positively correlated with income. However, Stephens et al. [25] found no significant differences in usage between private- and NHS-funded hearing aids. This suggests that whilst affordability might prove to be an initial barrier to the uptake of a hearing aid it has little bearing on continued use of the device. Other studies ([22], [29], [31], [50]), however, have reported that attitude towards rehabilitation, high cost, comfort, pre-fitting expectations and greater acceptance of hearing loss were found to be significantly related to hours of use.

## Discussion

Being able to determine whether a hearing aid is used regularly and effectively is an important measure of outcome. After all, what benefit is a hearing aid that provides improved speech intelligibility if it is never used? Equally, why wear a hearing aid if it does not provide any benefit? It has been recommended ([8], [51]) that regular follow up appointments in which the patient's progress with the device are required in order to improve patient outcomes. It is particularly important in older adults, where there is an increased risk of social isolation, that hearing loss does not go untreated; once a patient has been identified as having a hearing loss this invariably leads to the provision of hearing aids. The aim of this systematic review was to summarise the evidence available in the literature on how hearing aid usage in older adults has been measured in the last decade.

Our review identified fifteen different metrics for evaluating the usage of hearing aids; there was little consistency in the way that usage was recorded in the studies reviewed. Thus, it is fair to say that there is no standard tool for evaluating hearing aid usage. There was a dichotomy in the literature between those studies interested in the amount of time spent (i.e., hours per day) wearing a hearing aid, and those that focused on how regularly/frequently a hearing aid was used. Others used a mixed model asking what proportion of time a hearing aid was used in a specific situation. Tools like the IOI-HA [32] report a fixed number of hours which can be compared across listeners and studies fairly simply. The GHABP [15], on the other hand, takes a much more individualised approach which might better reflect the needs of the patient, but makes it very difficult to compare one patient with another. Both approaches have their merits and flaws so it is important to consider exactly which aspects of the patient's behaviour you are trying to capture.

One might have thought that distinguishing between ‘users’ and ‘non-users’ would be fairly straightforward, but this has not been the case. For instance, Gussekloo et al. [44] reported that 241 of 367 participants with severe hearing loss “did not make use of a hearing aid”. However, it was unclear from the text whether this response indicated “I do not own a hearing aid” or “I own a hearing aid, but do not wear it”. Similarly, the criteria for defining what constitutes ‘regular’ usage appeared somewhat arbitrary, as studies that employed such terminology tended not to specify the equivalent numerical cut-off point for these nominal categories. For this reason, we believe that having usage linked to a numerical value (e.g., hours per day or days per week) would prove to be extremely useful. The differences in data types (binary, ordinal, and interval) mean that it was impossible to calculate a definitive estimate of the proportion of patients that use their hearing aids or the amount of time that they spend wearing their hearing aids.

Typically, the success of hearing aid provision in the studies reviewed was determined by assessing how much benefit (e.g., objective and subjective ratings of speech intelligibility) a patient experienced. Our review suggests that the relationship between usage and other outcome domains is highly complex. Although a number of studies found that hearing aid usage was correlated with other outcome measures like benefit and satisfaction [9], [14], [28], [47] no single dimension was consistently shown to depend on the amount of time spent using a hearing aid. It is also noteworthy that while some patients may only require the use of hearing aids for specific situations and feel very satisfied, others may rely heavily on their hearing aids, using them throughout the day, but report low levels of satisfaction ([22], [29]).

We would like to reiterate arguments made in an earlier report [51] that recommended estimates of the time spent using a hearing aid should be cross validated. Ideally, this would involve a combination of objective (e.g., battery consumption or hearing aid data log) and subjective (e.g., diary or questionnaire) measures. If a numerical value of how many hours per day a patient wears a hearing aid (as suggested above) is not covered by a clinic's preferred self-report assessment tool then cross-validation would be one way of capturing that information. For instance, where the preferred clinical tool is the GHABP one might also consider including data from the hearing aid's data log. If an objective measure of usage is unavailable for cross validation, then a consensus approach using a second self-report questionnaire that does provide a numerical estimate of usage might be a reasonable solution. However, it should be remembered that self-reported usage measures are vulnerable to both over and under estimation (see [16], [24]).

While the usage of hearing aids does not guarantee successful patient outcomes, it is important to ensure that patients are using their hearing aids regularly and that the device makes a difference to the patient's ability to listen and communicate effectively. A number of studies reviewed asked patients how much they thought their life had changed as a result of a hearing aid, and many asked how much time was spent wearing hearing aids in particular situations. However, none of the outcome instruments identified in this review determined how much time was spent in that situation before receipt of a hearing aid. One might argue that a more useful measure of hearing aid outcome would be to determine how much *more* time people can spend doing the things they like as a result of wearing a hearing aid. It has been suggested that counselling [52] and motivational interviewing techniques [53] might improve not only the uptake of hearing aids, but also promote the continued use of the hearing instruments. Such a patient-centred, individualised approach paired with an objective measure of hearing aid usage in targeted situations might prove useful in planning and monitoring the auditory rehabilitation of patients receiving hearing aids.

Overall, we found that the level of reporting in the studies reviewed was inconsistent and of variable quality. There is a need for higher levels of evidence in the form of RCTs to study the impact of hearing aids as a treatment for hearing loss, and in particular for monitoring advances in service and technology on the uptake and compliance with hearing aid provision. Only with greater standardisation and precision in the level of reporting will future reviews of the literature be able to perform greater levels of analysis (e.g., meta-analysis) and provide firm guidelines on auditory habilitation with hearing aids.

In order to monitor auditory rehabilitation of patients provided with hearing aids it is important to determine that the patient is using the device regularly and effectively. Our review demonstrates that there is currently no standard method or format for reporting the amount of time spent using hearing aids. However, if one wants to be able to compare how much time is spent wearing hearing aids then outcome metrics do need to be standardised; a common metric would allow for meta-analysis and cross-comparisons to be undertaken.

While it is not likely, nor necessarily appropriate, that a single tool or method be adopted by clinical practices to evaluate compliance and success of hearing aid provision, it might be possible to reduce heterogeneity in reporting of usage data in research studies or health service evaluations by ensuring that when descriptive labels like regular and non-regular are to be used to characterize usage that they are clearly defined in terms of hours or days. Additionally, the assessment of hearing aid usage should be cross-validated by obtaining estimates with a number of different, preferably objective, assessment tools, and over multiple time courses in order to improve confidence in self-report estimates.

## Supporting Information

Table S1 **Summary of the data extraction from the 64 studies selected.** *Data estimated from figures HL: Hearing loss, PTA: Pure tone audiometry; sd: standard deviation; RCT = randomised control trial; OCS = observational case series; ROC = retrospective observational cohort.
(DOC)

Table S2 **Quality assessment and grading results.** Scoring codes are: 2 (study meets criterion to a high standard); 1 (study partially meets criterion); 0 (study does not meet criterion or relevant information is absent). The grading of the quality of evidence is: High (13–16), Moderate (8–12), and Low (4–7) and Very low (0–3) [19]. Abbreviations: Hearing loss (HL).
(DOC)
