# Supplementary material for: A Systematic Review of Studies Measuring and Reporting Hearing Aid Usage in Older Adults since 1999: A Descriptive Summary of Measurement Tools
Source: PLoS One. 2012 Mar 27;7(3):e31831. doi: 10.1371/journal.pone.0031831 (PMC3313982; doi:10.1371/journal.pone.0031831)
Supplement: Table S1 — Summary of the data extraction from the 64 studies selected. *Data estimated from figures HL: Hearing loss, PTA: Pure tone audiometry; sd: standard deviation; RCT = randomised control trial; OCS = observational case series; ROC = retrospective observational cohort. (DOC) [file pone.0031831.s001.doc]

| **Reference** | **Study design and aims** | **Sample size and sampling issues** | **Audiometry** | **Age** | **Follow up(s)** | **Outcome instruments** | **Findings** |
| --- | --- | --- | --- | --- | --- | --- | --- |
| **Arlinger & Billermark, 1999 [48]** | RCT to compare one-year follow up assessments of analogue and digital aids | N=29 Single centre. Preference to choose digital rather than analogue was controlled | Not reported | 45-77 years (mean 65) | 1 month and 1 year | APHAB [33]. The Gothenburg Profile [54]. Sound Quality Judgements [55]. Speech recognition in noise [56]. Structured interview | 89.7% 11 hrs/day. Amount of usage rose from 6 hours per day to 11 hours per day following transition from analogue to digital aids. Digital HA(s) were used almost twice as long than analogue HA(s) |
| **Banerjee, 2011 [57]** | OCS to examine the use of multi-memory and volume controls of hearing aids | N=9 One hearing aid clinic | PTA across ten frequencies (0.1-10khz). Mean data presented in figure | 49-78 yrs (mean 63.3, 10.8 sd) | Participant s were experienced hearing aid users, mean 11.9 yrs. 4-5 weeks | QuickSIN [37]. Log data | A total of 912 hrs (over 4-5 weeks) |
| **Baumfield & Dillon, 2001 [58]** | RCT to assess what factors contribute to use, benefit and satisfaction | N=29 Single centre | Mild-moderate (4 frequency average ranged from 26-59 dB HL) | 58-85 years (mean 71) | 3 and 6 weeks | Attitudes to hearing loss [59]. Speech recognition in noise [60]. GAS [61]. SHAPIE [62], [63]. HAUQ [64]. | Amount of usage not specified. Usage was associated with fitting accuracy, HA management, HA comfort, and style of fitting (eg, ITE/BTE) (*p*<0.05) |
| **Bertoli et al, 2009 [28]** | OCS to determine factors contributing to successful outcome | N=8707 One hearing centre with a market share of 20%. A random sample of non-respondents were contacted by phone (n=193) | Percentage of population with hearing loss at 4 frequencies | 15+ (proportion per decade is also given). Age is included in regression analyses | Participants could have received their hearing aid at any time in their life | Custom questionnaire. Battery consumption. IOI-HA [32] | 84.6% regular. 12.3% occasional. 3.1% never. Usage was associated with management of the HA, type of fitting (eg, binaural/monaural), type of HA(s) (eg, digital/analogue), age, audiometric data, experience with HA(s), gender, regional speech and satisfaction (*p*<0.05) |
| **Brannstrom & Wennerstrom, 2010 [42]** | OCS investigating the clinical application of Swedish translation of IOI-HA | N=224 One hospital. Demographic description of non-respondents | PTA Mean, sd and range | Mean 66.1 (27-94) | 6 months | IOI-HA [32] | Mean score 3.9 (1.1sd, 1-4 hr/day). Audiometric data, benefit, quality of life and satisfaction (*p*<0.05) |
| **Bratt et al, 2007 [27]** | Crossover clinical trial to investigate current use of study HAs, compare changes in audiological measures, and identify predictors of long-term HA use | N=210. 8 sites. Veterans and non-veterans. 38.1% female | Mean audiogram shown in figures | Mean 73.2 (range 36-96) | 5 to 7 years | NU-6 [65]. CST [36] [66]. Communication Profile for the hearing impaired [67]. Life Orientation Test (LOT) [68]. SADL [34] .PHAB [69]. GHABP [15]. IOI-HA [32]. HASQ [70] | Amount of usage not reported |
| **Chang et al, 2008 [71]** | OCS to evaluate speech performance differences in young and old elderly users of digital aids | N=59 One clinic. Observations made by the same audiologist | Means and sd | 65+ | 4 months | Speech reception thresholds (SRT). Most comfortable loudness level (MCL). COSI [72]. HHIE-S [73]. Custom questionnaire | 65-80 yrs group (N=32): 37.5% >8hr/day, 15.6% 5-8hr/day, 37.5% 1-4 hr/day, 94% <1hr/day. 80+ yrs group (N = 27): 33.3% >8hr/day, 14.8% 5-8hr/day, 33.3% 1-4 hr/day, 18.5% <1hr/day |
| **Collins et al, 2007 [74]** | ROC to further understand the effect of group visits on rehabilitation | N=74 Single clinic | PTA averages and sd for each ear for each group | Mean and sd for four groups: 65.9±11.9 67.9±8.8 66.8±8.9 67.3±9.7 | 90 days | HHIE [35]. SADL [34]. Effectiveness of auditory rehabilitation [75]. Custom questionnaire | Individual fittings = 10.2 hrs (sd 3.3). Group fittings = 12.4 hrs (sd 28). Usage was significantly greater in the group visit condition(*p*=002) |
| **Cook & Hawkins, 2007 [76]** | OCS to describe a useful patient report measure | N=262 Consecutive sample of new users from a single hearing clinic Response bias | Not specified | 73 (range 39-97) | Up to 12months | IOI-HA [32] | 79% at least 4hr/day |
| **Cox & Alexander, 2002 [77]** | OCS to report the psychometric properties of the IOI-HA for the original English version | N=172 Two private clinic. All individuals who purchased hearing aids during 1999 or 2000. Response rate 73% | Not reported | 26-98 yrs (mean 72) | 16% had worn their hearing aids for <3 months, 45% 3-12 months and 39% > 1 year | IOI-HA [32] | Mean score 4.1* (4-8 hrs/ day) |
| **Cox et al, 2003 [78]** | OCS to establish the norms for the IOI-HA | N=154. 80 clinics . 69% response rate | Three PTA across six frequencies (0.25-8kHz). Mean data presented in figure as a function of sex | Mean 77 yrs (10 sd) | 6-12 months | IOI-HA [32]. Custom questionnaire | Mean scores is given as a function of several factors like subjective hearing problems, HL, costs, etc. Usage ranged from 1-4 to 4-8 hrs/day |
| **Cox et al, 2007 [79]** | OCS to assess the relative contributions of patient variables and amplification variables on self-report outcomes | N=205 Eleven clinics. Veterans and non-veterans were recruited but number not balanced. Consecutive patients. No more than one participant per week was recruited at each site. Participation rate 85% | Mean audiogram shown in figure | Men 73 (sd 7.5) Female 75 (sd 7) | 6 months | NEO-FFI [80]. Response bias [81]. ECHO [82]. HHIE [73]. APHAB [69]. SHAPIE [62]. SADL [34]. Custom usage questionnaire | Mean 7.9 hrs/day (sd = 3.9 hrs) |
| **Cox et al, 2011 [83]** | OCS to predict which patients will prefer one hearing aid rather than two | N=91 Patients recruited from two sites via adverts, word of mouth, letters, etc. | PTA across seven frequencies (0.3-10kHz) shown in figure | 51-83 yrs (mean 69) | 9 weeks | IOI-HA [32]. APHAB [69]. DOSO [84]. ALDQ [15]. PANAS [85] | Mean score 4* (4-8 hrs/day approx.) |
| **Desjardins & Doherty, 2009 [86]** | OCS to assess experienced HA users' ability to use their HAs | N=50 Recruitment via radio and newspapers. Eleven clinics | 48.8 dB (sd 12.65). Mean audiogram shown in figure | Mean 75.36 (sd, 9.328) | 1 to 10+ years | SADL [34]. APHAB [69]. PHAST [86]. Custom questionnaire | 38% >12hr/day. 38% 6-12hr/day. 18% 2-5hr/day. 6% <2hr/day |
| **Dillon et al,1999 [14]** | OCS to provide normative data for the COSI and HAUQ questionnaires | N=4421 Participants randomly chosen from 46 different hearing centres. Clinicians (n=200) chose outcome measures. Response bias. Differences between hearing centres were assessed | % of patients per db loss category | Median 76 | 4-8 weeks and 3 months | APHAB [69]. COSI [59]. GAS [61]. HAUQ [64] | 34% >8hr/day*. 27% 4-8hr/day*. 28% 1-4hr/day*. 7% <1hrpd*. 2% <1hrpw*. 1% Never*. Usage was associated with: comfort, presence of feedback, quality of the user’s own voice (r=0.42). Benefit (r=0.43). Satisfaction (r=0.48) |
| **Gianopoulos et al, 2002 [87]** | OCS to examine the long-term use of HAs following a postal hearing screen | N=116 Four clinics. Data obtained from respondents and non-respondents was compared | Hearing level measured but not reported. | Age recorded but not reported | 8-16 years | SHHI [88]. ERS [89]. Interview | 43% regular. 57% never. Usage was associated with: management, size of HA, and style of fitting (eg, ITE/BTE) (effect size not reported) |
| **Gnewikow et al, 2009 [39]** | OCS to compare differences in omni-directional and directional microphones | N=105 Male veterans included in a single database from Tennessee Healthcare system. Does not specify how many clinics were involved | Mean audiogram shown in figure | Not reported | 1 month and 2 months | PHAB [69]. SADL [34]. Patient diary | All participants were previous wearers of binaural HAs with a minimum daily-usage of 4h/day. Participants were asked to wear the HA as much as possible during waking hours (a minimum of 6 h/day) |
| **Gopinath et al, 2011 [31]** | OCS to determine incidence and predictors of hearing aid use and ownership in older adults | N=1371 Population-based survey of age-related HL (Blue Mountains Hearing Study). Large community-based sample | PTA for 4 frequencies (0.5-4 kHz). Bilateral and unilateral degree of HL is presented as a function of hearing aid use or/and ownership | 55-99 years (mean 71.4) | 5 year | HHIE-S [90]. Structured interview covering medical history, demographics, education, lifestyle and hearing | Age, handicap and HL were significant predictors of incident hearing aid use/ownership. Usage only reported as a function of other factors |
| **Gussekloo et al, 2003 [43]** | OCS investigating a new screening test for older people | N=454 (367 with severe hearing loss). All 85+ year old inhabitants of Leiden. Demographic and health characteristics of those who participated and those who did not were compared | Median loss at 250 Hz and 8kHz | 85 | Not applicable | Custom questionnaire. MMSE [91]. GDS-15 [92]. Interview | 34% of the 367 were using a hearing aid at the time of the follow up |
| **Harkins & Tucker, 2007 [93]** | Internet survey to determine the use of HAs in a range of situations | N=423 Newsletters, conference flyers, e-mail announcements. Pilot studies were conducted. Acknowledgement of bias toward individuals who have knowledge of hearing technology | Percentage of population falling within each audiometric category | 18-81+  49% were aged 51-70 | One year | Interview | Not reported |
| **Hartley et al, 2010 [29]** | OCS to determine the prevalence, usage of hearing aids and assistive listening devices (ALD) | N=3956 Blue Mountain Hearing study. Population-based survey, 75.5% response rate | PTA across eight frequencies (0.25-8kHz). Degree of HL presented as a function of age, hearing aid and ALD ownership | 49-99 yrs (mean, 9.1 sd) | Not applicable | HHIE-S [90]. Custom questionnaire | Never 24.3%, <1hr/wk 7.4%, <1hr/day 7.4, 1-4hr/day 22.8, 4-8hr/day 12.7%, >8hr/day, 24.1%, Unsure 0.9% |
| **Heuermann et al, 2005 [94]** | OCS to compare the psychometric properties of the German IOI-HA version with previous studies | N=488 for mailing campaign. N=80 for field test. Non-respondent‘s demographics, HL and type of fitting were compared with respondents, no differences were found | Not reported | 19-95 yrs (mean 72) | At least more than 3 months before the mailing | IOI-HA [32]. Oldenburg Inventory [95]. Questionnaire filled by audiologists about the client’s hearing aid rating | Mailing campaign mean score 4.2 (1sd, 4-8 hrs/ day). Field test mean score 4.6 (0.7sd, 4-8 hrs/ day) |
| **Hickson et al, 1999 [50]** | OCS to determine the factors associated with outcomes | N=52 Consecutive sample of new users from one clinic (Australian Hearing Services-Queensland district). Possible response bias | Means and standard deviations given for each HL category | 63.2 (sd 8.97) range 60-97 | Two weeks and 3-9 months | HAUQ [64]. COSI [59]. Attitude type [59]. APHAB [69]. Custom questionnaire | 25% >8hr/day*. 40% 4-8hr/day*. 23% occasionally*. 12% <1hrpw or never*. Usage was associated with attitude towards rehabilitation (*p*<004) |
| **Hickson et al, 2010 [96]** | OCS to investigate factors associated with hearing aid fitting outcomes on the IOI-HA | N=1653, 15 clinics. Differences between respondents and non-respondents were asses, no differences were found | PTA across four frequencies (0.5-4kHz). Audiogram types (four groups) displayed in graph | 20-90+ yrs. 69% were age between 50-79 yrs | 6 months after fitting | IOI-HA [32]. EARtrak survey based on MarkeTrak V and VII [96], [98] | Mean score 4.1 (4-8 hrs/day) |
| **Hosford-Dunn & Halpern, 2000 [99]** | OCS to examine the validity of SADL | N=375 Consecutive series of patients from one clinic. Non-respondents were contacted | Average loss at each frequency given | Mean 76 (SD 12.8). Median 77 (range, 61-101) | Weekly for up to one year | SADL [34] | Usage not reported and values could not be estimated from figures |
| **Humes et al, 2001 [11]** | OCS to identify the domains of HA outcome | N=173 Recruitment via newspapers, flyers, printed announcements, and word to mouth | Mean audiogram shown in figure | Mean 73.1 (sd 6.5) | Two weeks, 1 month | CUNY NST [37]. CST [36], [66]. HAPI [62]. HHIE [73]. MarkeTrak-V hearing satisfaction survey [100]. SIR [101]. GHABP [15]. Patient diary. Hearing Activity Questionnaire | Participants wore their HA about ¾ of the time (mean score 4) across the four listening conditions of the GHABP |
| **Humes et al, 2002 [102]** | OCS to investigate changes in benefit over 2 years in older listeners | N=134 Recruitment via newspapers, flyers, printed announcements, and word to mouth | High frequency average | 72.6 (in year one) | Two weeks, 1 month, 12 months, 24 months | HHIE [73]. WASI-IQ [90]. CST [36], [66]. CUNY NST [37]. HAPI [62]. Patient diary | At fitting participants were instructed to use their HA for 4 hr/day and increase it to 6 hr/day after two weeks |
| **Humes et al, 2004 [47]** | RCT to compare benefit provided by different technologies/processing strategies | N=173 Recruitment via newspapers, flyers, printed announcements, and word to mouth | Mean audiogram shown in figure | Mean 75 | 1 month and 6 months | HAPI [62]. [73] HHIE. MarkeTrak-V hearing satisfaction survey [97]. CUNY NST [37]. CST [36], [66]. SPIN [60]. GHABP [15]. Patient diary. Hearing Disability and Aided Benefit Interview (HDABI) [15] | GHABP: Mean score of 3.5 out of 5*. Diary: 75 hr/day (sd = ~4)* . HDABI: 2hr/day* |
| **Humes et al, 2009 [46]** | RCT to compare differences in HA outcome of 4 groups of older adults with different HA technologies | N=333 Recruitment via newspapers, flyers, printed announcements, and word to mouth | Mean audiogram shown in figure | Mean and standard deviation is given for each of four groups  Mean=74.6 (sd 6.9) | Two weeks and 4-6 weeks | CST [36], [66]. HAPI [62]. MarkeTrak-IV hearing satisfaction survey [103]. Patient diary or daily log of usage. GHABP [15] | Diary or daily log: 7-8hr/day* (sd = 4 hrs). GHABP: Mean score of 4 out of 5. Usage was associated with Benefit and Satisfaction (effect size not reported) |
| **Ivory et al, 2009 [40]** | OCS to assess short-term (6 weeks) HA benefit | N=4584 Mostly older male Veteran medical centres from 2 States. Observations made from 48 audiologists from public and private sector | Mean, sd and range given for each frequency | Veterans - not reported | 6 weeks | Self-Assessment of Communication [104]. SAC-Hx [41] | Not reported |
| **Jerram & Purdy, 2001 [22]** | OCS to examine the influence of technology, demographics, expectations, attitudes and adjustment to hearing loss to HA outcomes | N=162 Patients from 12 private and 7 public hospitals | Mean audiogram shown in figure | Mean 70.5 (sd, 10.8) | One year | APHAB [69]. HARQ [105]. Expectations questionnaire [106]. CPHI ([67]. Custom questionnaire | 69% 4+ hrs day. 26% 1-4hr/day % <1hr/day. Usage was associated with: acceptance HL (*p*=0.048) and expectations (*p*=0.0004) |
| **Kam et al, 2011 [107]** | OCS to investigate the validity and reliability of the Chinese version of the APHAB | N=134 Participants recruited from the database of the Occupational Deafness Compensation Board | Mean PTA across four frequencies (0.5-4 kHz) 59.60 db HL (13.97 sd) and as function of age group | 52-87 (mean 67.5 yrs) | Mean hearing aid experience 3.03 yrs (0.62 sd) | APHAB-CH [107]. Questionnaire covering patterns of hearing aid use or hrs/day | 8% >16hr/day. 19% 8-16hr/day. 18% 4-8hr/day. 32% 1-4hr/day. 6% <1hr/day. 7% None |
| **Keidser et al, 2008 [49]** | ROC to investigate the long-term benefit of digital HA | N=30 Original sample was recruited from database of volunteers, community-based hearing help organisations and advertisements in the local paper | PTA 0.5-2kHz. Group one: 78.1 dB. Group two: 72.4dB. Details in table | Mean Group one: 59.3 Group two: 65.7. Details in table. | Group one at 6 months, Group two at 24 months | Custom questionnaire | Figures show daily usage of between 1-4 and >8 hours. Usage was associated with Satisfaction (*p*<005) |
| **Kemker & Holmes, 2004 [108]** | RCT to evaluate the effect of the pre-fitting and post-fitting hearing aid orientation sessions | N=45 One clinic. Two experimental groups + control. Systematic random sampling scheme | PTA across three frequencies (0.5-2kHz), reports mean and sd per group | 70.7 yrs (60-80) | 5 weeks | GHABP [15] | Mean, sd, median and max/min values are given per group. Mean scores control 4.42, Prefit 4.54 and postfit 4.34 (3/4 of the time) |
| **Kricos et al, 2007 [109]** | OCS to investigate the psychosocial correlates of HA use | N=197 Patients in the National Institute on Deafness and Other Communication Disorders/Veterans Affairs | Information given in Bratt 2007). | 36 to 96 yrs (mean 73.2) | 5 to 7 years | NU-6 [65]. Monosyllabic word-recognition test [110]. CST [36], [66]. SADL [34]. PHAB [69], LOT [68], GHABP [15]. Interview | Amount of Usage reported in companion paper [129]. Usage was associated with: Challenging life events (*p*<0.05) |
| **Liu et al, 2011 [111]** | OCS to present normative data on the Chinese version of the IOI-HA and assess the service effectiveness | N=1049, 14 clinics | Mean PTA across six frequencies (0.25-8kHz) displayed in graph | 18-93 yrs (mean 60.7) | At least more than 3 months post-fitting | IOI-HA [32]. Custom questionnaire for demographic s and information about the hearing aid | Mean score 4.2* (0.96 sd, 4-8 hrs/day) |
| **Lockey et al, 2010 [112]** | Inductive qualitative study using interpretative phenomenology to learn more about consistent HA users to promote use in non-users | N=4 Women recruited by 2 mall walkers | Not reported | 80, 83, 69, 61 | Year of HA use 20, 6, 5, 30 | Biographic-narrative-interview [138] | Participants reported wearing hearing aid: when they leave the house, from moment wakes up till goes to bed, when interacting with others, when they leave the house. Usage was associated with: Meaningful participation in life (effect size not reported) |
| **Lupsako & Kautiainen, 2005 [23]** | OCS to investigate the determinants of non-use of HAs | N=601 Randomly selected from the register of Kuopio. Response bias. Adjustments for multiple (post hoc) testing | Not reported | 81.3 (sd = 5) | Not reported | Custom questionnaire. MMSE [91]. DSI [113]. ADLs [114] | 55% full time. 20% part-time. 25% Never. Usage was associated with: Cognitive decline (*p*=0.006), income (*p*=0.002), and impact of HL in daily activities (*p*=0.005) |
| **Maki-Torkko et al, 2001 [24]** | OCS to investigate objective and subjective measures of HA usage | N=84 Consecutive series of patients from two hospitals. Possible response bias | Not reported | 17 to 81 (median 54) | 21-73 days | Custom questionnaire. Patient diary. HA data log. Interview | Data log: 35.7% >8 hr/day. 29.8% 4-8hr/day, 34.5% <4hr/day. Diary: 32.1% >8 hr/day, 25% 4-8hr/day, 29.8% <4hr/day. Interview: 42.9% >8 hr/day, 34.5% 4-8hr/day, 22.6% <4hr/day |
| **Meister et al, 2005 [115]** | OCS to detect the factors underlying successful HA fittings/outcomes | N=150 Two sites (public and private). Participants randomly recruited | Mean thresholds at 4 frequencies given | Mean 64 (range 16-92) | Not reported | Custom questionnaire | Average score of about 8 on an 11-point scale (never-to-always). Usage was associated with: HA performance, acceptance HL, attitude towards rehabilitation, expectations, benefit, handicap and satisfaction (*p*<0.05) |
| **Munro & Lutman, 2004 [116]** | OCS to assess self-reported outcomes in new HA users 24 weeks post-fitting | N=32 Local hospital audiology services | Full range given in mean and sd | 74 (sd 5.4) | 24 weeks | GHABP [15] | Not reported |
| **Olusanya, 2004 [117]** | OCS to evaluate self-reported outcomes among HA users | N=99 Single centre | Percentage of participants with losses in each HL category (i.e., mild, moderate or severe) | 16-89 (mean 45.8) | Patients with <3, 3-12 or >12 months experience were tested | IOI-HA [32] | 51% >8hr/day, 21%* 4-8hr/day, 16%* 1-4hr/day, 7%* <1hrpw, 5%* Never. Usage was associated with: Impact of HL in daily activities, benefit, and quality of life *p*<0.05) |
| **Parving & Christensen, 2004 [118]** | OCS to assess the clinical outcome of an analogue, low cost, behind-the-ear hearing aid | N=25 One clinic | Median hearing thresholds and ranges are presented in a table across six frequencies (0.25-8kHz) | 50-86 yrs (mean 74) | 6 weeks after fitting | IOI-HA [32]. Speech recognition in background noise | Median score 4 for the test hearing aid (4-8 hrs/ day). Median score 5 for the non-test hearing aid (>8hr/day) |
| **Parving & Sibelle, 2001 [45]** | OCS to document longitudinal improvements/changes in hearing instrument benefit, use and satisfaction | N=32694 All National Hearing Health Services users in Denmark. Demographic and hearing instrument distribution of those who participated and those who did not were compared | Not reported | Median 78 (range 18-102) | 3-4 months | Custom questionnaire | 85%* Daily/Weekly, 5%* Seldom/Never |
| **Purdy & Jerram, 2001 [119]** | OCS to evaluate a shortened version of the PHAP [120] - the APHAP | N=67  University database and participants from previous study | Better-ear four frequency average | Mean 64.9 (sd 12.7)  range 26 to 88 | Not applicable | PHAP [120]. Custom questionnaire | 10.9 hr/day (sd = 47hr). Usage was associated with: Degree of HL (*p*=0.001), satisfaction (*p*=0.001) and HA performance (*p*<0.006) |
| **Reber & Kompis, 2005 [121]** | RCT to study auditory acclimatization and outcome in first-time hearing aid users | N=23 Single clinic and single audiologist. Single audiologist delivering different fitting protocols | Mean audiogram shown in figure for each group. Group 1: Normal hearing <2kHZ. Group 2: Normal hearing <1kHZ | Mean 64 (range 40-76) | 2 weeks and 3 months | Custom questionnaire | Average usage +/- 1 sd given in figure for the three treatment groups. Usage was associated with: Fitting protocol (eg, patient/audiologist driven) (*p*<0.01) |
| **Roup & Noe, 2009 [10]** | OCS to investigate the benefit of completely in the canal HAs for listeners with high-frequency losses | N=53 Single audiology clinic for veterans. Non-respondents were contacted | Mean audiogram shown in figure for each group. Group 1: Normal hearing <2kHZ. Group 2: Normal hearing <1kHZ | Group 1: Mean 53 (range 38-75). Group 2 mean 63 (range, 51-79). | Within 4 years | HHIE-S [90]. APHAB [69]. SADL [34] | 15% 8-16hr/day*. 27% 4-8hr/day*. 50% 1-4hr/day*. 8% <1hr/day*. Employed HA users wore HA(s) more often than retired users (effect size not reported) |
| **Schneider et al, 2010 [5]** | OCS to examine the impact of hearing loss on the use of community and informal supports | N=2956. Blue Mountains Hearing Study Cohort | PTA (0.5-4 kHz) 33% sample >25dB HL | Group “No HL” mean: 64.3 (7.9 sd). Group “HL” mean: 73.7 (8.4 sd) | Current users and 5 years follow up | MMSE [91]. Interview | Usage data not reported. Frequent use of hearing aid was defined as >1h/day and infrequent as <1h day. Usage was associated with: the use of community support services |
| **Shanks et al, 2007 [122]** | OCS to examine long-term HA benefit over a six-year period | N=108 Eight veterans centre and private sector. Private sector was included to make results applicable to a broader range of HA users | Means and sd of users and non-users PTA given in a figure | 73.2 | 5 to 6 years | NU-6 [65]. CST [36], [66]. Custom questionnaire | 85.2% current users. 14.8% currently non-users |
| **Smeeth et al, 2002 [26]** | OCS to assess the prevalence of reduced hearing in elderly people and levels of ownership and use | N=32656 , 3846 owned a hearing aid. 106 family practices in the UK. Possible response bias. Practices were selected by standardised mortality ratio | Pass and fail on the whispered voice test is reported | 75+ The number of people in increasing 4-year age brackets is reported | Time between receiving an aid and completing question is not specified. Assessments were completed between 1995 and 1999 | Custom questionnaire [123]. Whispered voice test [126] | 60% Regular, 40% Never. Usage was associated with : Gender (*p*=0047) and benefit (*p*=00001) |
| **Smith et al, 2009 [124]** | OCS to evaluate the psychometric properties of the IOI-HA [32] | N=131 Patients randomly selected from a clinic, 52.4% response rate | PTA across six frequencies (0.25-8kHz), man shown in figure | 55-87 yrs (mean 74.3, sd 7.4) | >6 months but > 2 yrs | IOI-HA [32] | Mean score 3.6 (1-4hrs/day) |
| **Stark & Hickson, 2004 [125]** | OCS to examine the effect of hearing impairment on the patient's and the significant other's quality of life | N=131, 93 after drop outs, failure to return follow up questionnaires etc). One audiology clinic. Possible response bias | The number of people with hearing loss at various levels is reported | 47-90 (mean 71.7, sd 8.6). | 2 weeks | HHIE [73], Attitude type [59]. QDS [104]. Custom questionnaire | 14% >8hr/day. 28% 4-8hr/day. 31.2% 1-4hr/day. 15.1% >1hr/week & <1hr/day. 75% <1hr/week. 4% Never |
| **Stephens et al, 2001 [25]** | OCS to examine changes in hearing aid possession and use over an 18 year period | N=2632 Participants randomly selected according to their postal code or from a Welsh Office list of those elderly who had spent at least 24hr in an acute hospital. Possible response bias | Not reported | 65+ | Not applicable | MRC NSH and ENT questionnaire [1], [127]. Custom questionnaire | 56.8% most of the time. 26.3% some of the time. 15.8% no longer use hearing aid. Usage was associated with: Age (effect size not reported) |
| **Stephens, 2002 [128]** | OCS to understand the relationship between the IOI-HA and the COSI | N=161 Consecutive patients from a single clinic during 2000-01 | PTA (0.5-4kHz) mean 38.8 dB (14.3sd) | 40-94 yrs (mean 72.4, 10.9 sd) | Not specified | IOI-HA [32]. COSI [59] | 3.7 (0.86 sd, 1-4 hrs/day) |
| **Takahashi et al, 2007 [129]** | OCS to examine perceived benefit and satisfaction in a six year period | 164 for GHABP but varied for other measures. Veterans and non-veterans. 8 sites. 38.1% female | See [27] | Mean 73.2 (range 36-96) | 6 years | HASQ [70]. PHAP [120]. PHAB [69]. SADL [34]. GHABP [15]. IOI-HA [32] | GHABP: (mean use 4.3 sd 1). See Fig 11 for breakdown of usage in each scenario. IOI_HA: 66% >8hr/day*, 12% 4-8hr/day*, 19% 1-4hr/day*, 3% <1hrpw* |
| **Taubman et al, 1999 [16]** | RCT to investigate the accuracy of self-reported usage estimates | N=24 | Pure tone average and sd for experimental and control group | Mean 70.8, range 50 to 90 | 1 week | SIR [101]. HA data log. APHAB [69] | Data log: 79% >8hr/day, 8% 4-8hr/day, 9% Occasionally, 4% <1hrpw. APHAB: 72%>8hr/day, 16% 4-8hr/day, 10%. Occasionally, 2% <1hrpw |
| **Tomita et al, 2001 [130]** | OCS to investigate how assistive technology can be used by older people | N=227 Possible response bias. Sample of elderly that participated in a previous study. This sample resembled the national population | Not reported. | 76.7 (sd 8.1) range 65+ | Not applicable | CAS interview battery [131]. Custom questionnaire | 68.7% of participants with hearing impairment did not use HA |
| **Uriarte et al, 2005 [9]** | OCS to investigate HA satisfaction using SADL and other outcome measures | N=1014 Sample of new and return clients fitted with a government-funded HA on a pension. Possible response bias. 8% of the target population was randomly sampled from each Australian state and territory | Mean better-ear three-frequency average = 40.21 sd (14.46) | Mean 75.32 years, sd 9.73, range 29-104 | Various levels of experience prior to receiving questionnaires | SADL [34]. CSS [14]. Custom questionnaire | 30% >8hr/day*, 26% 5-8hr/day*, 35% 1-4hr/day*, 5% <1hrpd*, 2% <1hrpw*, 3% Never. Usage was associated with: Satisfaction (p<0.001) |
| **Vestergaard, 2006 [132]** | OCS to compare changes in self-report scores over time using various outcome measures | N=25 Single clinic | Steeply sloping loss with mean 55dB/oct (sd17db/oct) | 60.4 (sd 10.8) | Various levels of previous experience. Outcomes conducted at 1 week, four weeks and 13 weeks | SADL [34]. GHABP [15]. IOI-HA [32] | Not reported |
| **Vuorialho et al, 2006 [44]** | OCS to investigate how the use of HAs is affected by changes in service and technology and society over 20 years | N=76 Consecutive patients from one hospital. Possible response bias. Comparisons were made with the general elderly population | Not reported | Median 73.8 | 6 months | Interview | 56.6% regular (>2hr/day). 36.8% occasional (<1hr/day). 5.3% non-users. 1.3% missing data |
| **Vuorialho et al, 2006 [52]** | OCS to assess the cost and the effect of follow-up counselling on HA use | N=98 Consecutive patients from one hospital. Possible response bias | Not reported | 76.7 range (47 to 87) | 6 and 12 months | HHIE [73]. EuroQoL [133]. Interview | At 6 months: 61.2% regular (>2hr/day), 29.6% occasional (<1hr/day), 9% non-users. At 12 months: 77.6% regular, 17.3 occasional, 5.1% non-users. Usage was associated with: Follow-up counselling (*p*<001) |
| **Walden & Walden, 2004 [134]** | OCS to investigate the relationship between various demographic and audiometric measures and HA outcome | N=50 Single army clinic. Consecutive patients | Mean audiogram shown in figure | Mean 72.1 (sd 12.3) range, 49-94) | 195 months of experience wearing hearing aid | IOI-HA [32] | Mean 8.6 hrs/day (sd = 4.2). Usage was associated with: Degree of HL (*p*<0.01), Unaided articulation index (*p*<0.05), benefit (*p*<0.01), and satisfaction (p<0.01) |
| **Williams et al, 2009 [135]** | OCS to explore the effects of gender and experience on self-report scores | N=64 Consecutive patients from two hearing clinics Possible response bias | PTA across five frequencies (0.5-8kHz), mean shown in figure | Mean 73 (22-94) | At least 3 months | Custom questionnaire. IOI-HA [32] | Mean score 4.4 (4-8 hrs/day). 61% >8hr/day. 22% 4-8hr/day. 14% 1-4hr/day. 3% <1hrpw |
| **Yueh et al, 2010 [136]** | RCT to assess the effect of hearing screening on outcomes | N=2314  Recruited through flyers and posters in streets  Veterans with 50% or greater service-connected disability ratings  Non-respondents were contacted | Collected but not displayed in the paper | Mean 60.7 | One year | HHIE-S [90], VR-36 [137], Inner EAR [75], APHAB [69] | Not reported |

**References Table S1**

1. Davis AC, Smith P, Ferguson M, Stephens D, Gianopoulus I (2007) Acceptability, benefit and costs of early screening for hearing disability: a study of potential screening tests and models. Health Technol Assess 11: 3-5.

5. Schneider J, Gopinath B, Karpa M, McMahon C, Rochtchina E, et al. (2010) Hearing loss impacts on the use of community and informal supports. Age Ageing 39: 458-464.

9. Uriarte M, Denzin L, Dunstan A, Sellars J, Hickson L (2005) Measuring hearing aid outcomes using the Satisfaction with Amplification in Daily Life (SADL) questionnaire: Australian data. J Am AcadAudiol 16: 383-402.

10. Roup C, Noe C (2009) Hearing aid outcomes for listeners with high-frequency hearing loss. Am J Audiol, 18: 45-52.

11. Humes L, Garner C, Wilson D, Barlow N (2001) Hearing aid outcome measures following one month of hearing aid use by the elderly. J Speech Hear Res: 443: 469-486.

14. Dillon H, Birtles G, Lovegrove R (1999) Measuring the outcomes of a national rehabilitation program: Normative data for the client oriented scale of improvement (COSI) and the hearing aid user's questionnaire (HAUQ). J Am AcadAudiol 10: 67-79.

15. Gatehouse S (1999) Glasgow hearing aid benefit profile: derivation and validation of a client-centered outcome measure for hearing aid services. J Am AcadAudiol 10: 80-103.

16. Taubman L, Palmer C, Durrant J, Pratt S (1999) Accuracy of hearing aid use time as reported by experienced hearing aid wearers. Ear Hear 20: 299-305.

22. Jerram JC, Purdy SC (2001) Technology, expectations, and adjustment to hearing loss: predictors of hearing aid outcome. J Am AcadAudiol 12: 64-79

23. Lupsakko T, Kautiainen H, Sulkava R (2005) The non-use of hearing aids in people aged 75 years and over in the city of Kuopio in Finland. Eur Arch Otorhinolaryngol 262: 165-169.

24. Maki-Torkko E, Sorri M, Laukli E (2001) Objective assessment of hearing aid use. ScandAudiol, 30: 81-82.

25. Stephens D, Lewis P, Davis A, Gianopoulos I, Vetter N (2001) Hearing aid possession in the population: Lessons from a small country. Audiology 40: 104-111.

26. Smeeth L, Fletcher A, Ng ESW, Stirling S, Nunes M, et al. (2002) Reduced hearing, ownership, and use of hearing aids in elderly people in the UK - the MRC Trial of the Assessment and Management of Older People in the Community: a cross-sectional survey. Lancet 359: 1466-1470.

27. Bratt G, Rosenfield M, Williams D (2007) NIDCD/VA hearing aid clinical trial and follow-up: Background. J Am AcadAudiol 18(4): 274-281.

28. Bertoli S, Staehelin K, Zemp E, Schindler C, Bodmer D, et al. (2009) Survey on hearing aid use and satisfaction in Switzerland and their determinants. Int J Audiology 48: 183-195.

29. Hartley D, Rochtchina E, Newall P, Golding M, Mitchell P (2010) Use of Hearing Aids and Assistive Listening Devices in an Older Australian Population. JAm AcadAudiol, 21(10): 642-653.

31. Gopinath B, Mitchell P, Schneider J, Hartley D, Teber E, et al. (2011) Incidence and Predictors of Hearing Aid Use and Ownership among Older Adults with Hearing Loss. Annals of Epidemiology 21(7): 497-506.

32. Cox R, Hyde M, Gatehouse S, Noble W, Harvey D, et al. (2000) Optimal outcome measures, research priorities, and international cooperation. Ear Hear 21(4 Suppl): 106S-115S.

33. Cox R, Alexander G (1995) The abbreviated profile of hearing aid benefit. Ear Hear 16: 176-186.

34. Cox R, Alexander G (1999) Measuring satisfaction with amplification in daily life: the SADL scale. Ear Hear 20: 306-320.

35. Ventry I, Weinstein B (1982) The Hearing Handicap Inventory for the Elderly: a new tool. Ear and Hearing 3(3): 128-34.

36. Cox R, Alexander G, Gilmore C, Pusakulich K (1988) Use of Connected Speech Test (CST) with hearing-impaired listeners. Ear Hear 9: 198-207.

37. Levitt H, Resnick S (1978) Speech reception by the hearing impaired: Methods of testing and development of meterials. Scand Audiol Suppl 6: 107-129.

39. Gnewikow D, Ricketts T, Bratt G, Mutchler L (2009) Real-world benefit from directional microphone hearing aids. J Rehabil Res Dev 46(5): 603-618.

40. Ivory P, Hendricks B, Van Vliet D, Beyer C, Abrams H (2009) Short-term hearing aid benefit in a large group. Trends Amplif 13(4): 260-280.

42. Brannstrom K J, Wennerstrom I (2010) Hearing aid fitting outcome: clinical application and psychometric properties of a Swedish translation of the international outcome inventory for hearing aids (IOI-HA). J Am Acad Audiol, 21(8): 512-521.

43. Gussekloo J, de Bont LE, von Faber M, Eekhof J, Laat J, et al. (2003) Auditory rehabilitation of older people from the general population--the Leiden 85-plus study. Br J Gen Pract 53: 536-540.

44. Vuorialho A, Sorri M, Nuojua I, Mulhi A (2006) Changes in hearing aid use over the past 20 years. Eu Arch Oto-Rhino-Laryng 263: 355-360.

45. Parving A, Sibelle P (2001) Clinical study of hearing instruments: A cross-sectional longitudinal audit based on consumer experiences. Audiology 40: 43-53.

46. Humes L, Alhstrom J, Bratt G, Peek B (2009) Studies of hearing-aid outcome measures in older adults: A comparison of technologies and an examination of individual differences. Sem Hear 30: 112-128.

47. Humes L, Humes L, Wilson D (2004) A comparison of single-channel linear amplification and tow-channel wide-dynamic-range-compression amplification by means of an independent-group design. Am J Audiol 13: 39-53.

48. Arlinger S, Billermark E (1999) One year follow-up of users of a digital hearing aid. Br J Audiol, 33: 223-232.

49. Keidser G, Hartley D, Carter L (2008) Long-term usage of modern signal processing by listeners with severe or profound hearing loss: a retrospective survey. Am J Audiol 17: 136-146.

50. Hickson L, Timm M, Worrall L, Bishop K (1999) Hearing aid fitting: outcomes for older adults. Aust J Audiology 21(1): 11.

52. Vuorialho A, Karinen P, SorriM (2006) Effect of hearing aids on hearing disability and quality of life in the elderly. Int J Audiol 45(7): 400-405.

54. Ringdahl A, Erickson M, Karlsson K (1998) Psychometric evaluation of the Gothenburg profile for measurement of experienced hearing disability and handicap with new hearing aid candidates and experienced hearing aid users. Br J Audiol 32: 375-385.

55. Gabrielson A, Schenkman BA, Hagerman B (1988) The effects of different frequency responses on sound quality judgements and speech intelligibility. J Acoust Soc Am 31: 166-177.

56. Hagerman B, Kinnefors C (1995)Efficient adaptive methods for measurements of speech reception thresholds in quiet and in noise. Scand Audiol 24: 71-77.

57. Banerjee S (2011) Hearing aids in the real world: use of multimemory and volume controls. J Am Acad Audiol 22(6): 359-374.

58. Baumfield A, Dillon H (2001) Factors affecting the use and perceived benefit of ITE and BTE hearing aids. Br J Audiol 35(4): 247-258.

59. Goldstein D, Stephens SD (1981) Audiological rehabilitation: management model I. Audiology 20: 432-452.

60. Kalikow DN, Stevens K, Elliott L (1977) Development of a test of speech intelligibility in noise using sentence material with controlled word predictability. J Acoust Soc Am 61: 1337-1351.

61. Dillon H, Koritschoner E, Battaglia J, Lovegrove R, Ginis J, et al. (1991) Rehabilitation effectiveness I: Assessing the outcomes for clients of a national hearing rehabilitation program. Aust J Audiology 13: 68-82.

62. Walden B, Demorest M, Hepler E (1984) Self-report approach to assessing benefit derived from amplification. J Speech Hear Res 27: 49-56.

63. Dillon H (1994) Shortened hearing aid performance inventory for the elderly (SHAPIE): a statistical approach. Aust J Audiology 16: 37-48.

64. Forster S, Tomlin A (1988) Hearing Aid Usage in Queensland. Paper presented at the Audiological Society of Australia Conference.

65. Tillman T, Carhart R (1966) An Expanded Test for Speech Discrimination Utilizing CNC Monosyllabic Words. Northwestern University Auditory Test No. 6: Brooks Air Force Base, TX: USAF school of Aerospace Medicine.

66. Cox R, Alexander G, Gilmore C (1987). Development of the connected speech test (CST). Ear Hear 8: 119S-126S.

67. Demorest M, Erdman S (1987) Development of the communication profile for the hearing impaired. J Speech Hear Res 52(2): 129-139.

68. Scheir M, Carver C (1985) Optimism, coping, and health: assessment and implications of generalized outcomes expectations. Health Psychol 54: 1063-1070.

69. Cox R, Rivera I (1992) Predictability and reliability of hearing aid benefit measured using the PHAB. J Am Acad Audiol 3(4): 242-254.

70. Boothroyd A, Noffsinger D (2001) Hearings status questionnaire (HASQ).

71. Chang W H, Tseng HC, Chao TK, Hsu C, Liu T (2008) Measurement of hearing aid outcome in the elderly: Comparison between young and old elderly. Otolaryngol Head Neck Surg 138(6): 730-734.

72. Dillon H, James A, Ginis J (1997) Client Oriented Scale of Improvement (COSI) and its relationship to several other measures of benefit and satisfaction provided by hearing aids. J Am Acad Audiol 8(1): 27-43.

73. Weinstein B (1986) Validity of a screening protocol to identifying elderly people with hearing problems. ASHA 28: 41-45.

74. Collins MP, Souza PE, O'Neill S, Yueh B (2007) Effectiveness of group versus individual hearing aid visits. J Rehabil Res Dev 44(5): 739-749.

75. Yueh B, McDowell JA, Collins M, Souza P, Loovis C, et al. (2005) Development and validation of the effectiveness of corrected auditory rehabilitation scale. Arch Otolaryngol Head Neck Surg 131(10): 851-856.

76. Cook J, Hawkins D (2007) Outcome measurements for patients receiving hearing aid services. The Laryngoscope 117: 610-613.

77. Cox RM, Alexander GC (2002) The International Outcome Inventory for Hearing Aids (IOI-HA): psychometric properties of the English version. Int J Audiol 41(1): 30-35.

78. Cox RM, Alexander GC, Beyer CM (2003) Norms for the international outcome inventory for hearing aids. J Am Acad Audiol 14(8): 403-413.

79. Cox R, Alexander G, Gray G (2007) Personality, hearing problems, and amplification characteristics: contributions to self-report hearing aid outcomes. Ear Hear 28(2): 141-162.

80. Costa P, McCrae R (1997) Longitudinal stability in adult personality. In R. Hogan, J. Johnson & S. Briggs (eds.), Handbook of Personality. Psychology San Diego, CA: Academi Press, pp. 269-290.

81. Hays R D, Hayashi T, Stewart AL (1989) A 5-Item Measure of Socially Desirable Response Set. Educational and Psychological Measurement 49(3): 629-636.

82. Cox R, Alexander G (2000) Expectations about hearing aids and their relationship to fitting outcome. J Am Acad Audiol 11(7): 368-382.

83. Cox RM, Schwartz KS, Noe CM (2011) Preference for One or Two Hearing Aids among Adult Patients (vol 32, pg 181, 2011). Ear Hear 32(3): 409-409.

84. Cox R, Alexander G, Xu J (2009) Development of the Device Oriented Subjective Outcome Scale (DOSO). Annual Meeting of the American Auditory Society. http://www.memphis.edu/ausp/harl/publications.htm#posters.

85. Watson D, Clark LA, Tellegen A (1988) Development and validation of brief measures of positive and negative affect: the PANAS scales. J Pers Soc Psychol 54(6): 1063-1070.

86. Desjardins J, Doherty K (2009) Do experienced hearing aid users know how to use their hearing AIDS correctly? Am J Audiol 18(1): 69-76.

87. Gianopoulos I, Stephens D, Davis A (2002) Follow up of people fitted with hearing aids after adult hearing screening: the need for support after fitting. BMJ 325: 471-471.

88. Rosen J (1979) An evaluation of the English language version of the social hearing handicap index. Scand Audiol 9: 111-115.

89. Noble W, Atherley G (1970) Hearing Measure Scale - Questionnaire for Assessment of Auditory Disability. Journal of Auditory Research 10(3): 229-250.

90. Wechsler D (1981) The Wechsler Adult Intelligence Scale-Revised. New York: The Psychological Corporation.

91. Tombaugh T, McIntyre N (1992)The mini-mental state examination: a comprehensive review. J Am GeriatrSoc 40: 922-935.

92. Yesavage J, Brink T, Rose T, Lum O, Huang V, et al. (1982) Development and validation of a geriatric depression screening scale: a preliminary report. J Psychiatr Res 1: 37-49.

93. Harkins J, Tucker P (2007) An internet survey of individuals with hearing loss regarding assistive listening devices. Trends Amplif 11(2): 91-100.

94. Heuermann H, Kinkel M, Tchorz J (2005) Comparison of psychometric properties of the International Outcome Inventory for Hearing Aids (IOI-HA) in various studies. Int J Audiol 44(2): 102-109.

95. Holube I, Kollmeier B (1994)ModifikationeinesFragebogenszurErfassung des subjektivenHorvermogens und dessenBeziehungzurSprachverstandlichkeit in Ruhe und unterStorgerauschen. AudiolAkust 33: 22-35.

96. Hickson L, Clutterbuck S, Khan A (2010) Factors associated with hearing aid fitting outcomes on the IOI-HA. Int J Audiol 49(8): 586-595.

97. Kochkin S (2000) MarkeTrak V: Consumer satisfaction revisited. Hear J 53(1): 38-55.

98. Kochkin S (2005) MarkeTrak VII: Customer satisfaction with hearing instruments in the digital age. Hear J 58(9).

99. Hosford-Dunn H, Halpern J (2000) Clinical application of the satisfaction with amplification in daily life scale in private practice I: statistical, content, and factorial validity. J Am Acad Audiol 11(10): 523-539.

100. Kochkin S (1997) MarketTrak V: What is the viable Market for Hearing Aids? Hearing J 50: 5.

101. Cox R, McDaniel D (1989) Development of the Speech Intelligibility Rating (SIR) test for hearing aid comparisons. J Speech Hear Res 32: 347-352.

102. Humes L, Wilson D, Barlow N, Barlow N (2002) Changes in hearing-aid benefit following 1 or 2 years of hearing-aid use by older adults. J Speech Hear Res 45(4): 772-782.

103. Kochkin S (1996) MarkeTrak IV: 10-year trends in the hearing aid market -has anything changed? Hearing J 49: 1-6.

104. Schow R, Nerbonne M A (1982) Communication screening profile; use with elderly clients. Ear Hear 3: 135-147.

105. Hallam R, Brooks D (1996) Development of the Hearing Attitudes in Rehabilitation Questionnaire. Br J Audiol 30: 199-213.

106. Seyfried D (1990) Use of a Communication Self-Report Inventory to Measure Hearing Aid Counselling Effects. Unpublished Doctoral Thesis.University of Iowa.

107. Kam AC, Tong MC, van Hasselt (2011) Cross-cultural adaptation and validation of the Chinese abbreviated profile of hearing aid benefit. Int J Audiol 50(5): 334-339.

108. Kemker BE, Holmes AE (2004) Analysis of prefitting versus postfitting hearing aid orientation using the Glasgow Hearing Aid Benefit Profile (GHABP). J Am AcadAudiol 15(4): 311-323.

109. Kricos P, Erdman S, Bratt G, Williams D (2007) Psychosocial correlates of hearing aid adjustment. J Am Acad Audiol 18(4): 304-322.

110. Wilson R (1993) Development and use of auditory compact discs in auditory evaluation. J Rehabil Res Dev 30: 342-351.

111. Liu H, Zhang H, Liu S, Chen X, Han D, Zhang L (2011) International outcome inventory for hearing aids (IOI-HA): Results from the Chinese version. Int J Audiol 50(10): 673-678.

112. Lockey K, Jennings MB, Shaw L (2010) Exploring hearing aid use in older women through narratives. Int J Audiol: 49(8): 542-549.

113. Wade D, Collin C (1988) The Barthel ADL index: a standard measure of physical disability? Int Disabil Stud 10: 64-67.

114. Zung W (1972) The depression status inventory: an adjunct to the self-rating depression scale. J Clinic Psychology 28: 539-543.

115. Meister H, Lausberg I, Kiessling J, von Wedel, H, Walger M (2005) Detecting components of hearing aid fitting using a self-assessment-inventory. Eur Arch Otorhinolaryngol 262(7): 580-586.

116. Munro K, Lutman M (2004) Self-reported outcome in new hearing aid users over a 24-week post-fitting period. Int J Audiol 43(10): 555-562.

117. Olusanya B (2004) Self-reported outcomes of aural rehabilitation in a developing country. Int J Audiol 43(10): 563-571.

118. Parving A, Christensen B (2004) Clinical trial of a low-cost, solar-powered hearing aid. Acta Otolaryngol 124(4): 416-420.

119. Purdy S, Jerram J (2001) Investigation of the profile of hearing aid performance in experienced hearing aid users. Ear Hear 19(6): 473-480.

120. Cox R, Gilmore C (1990) Development of the Profile of Hearing Aid Performance (PHAP). J Speech Hear Res 33(2): 343-357.

121. Reber M, Kompis M (2005) Acclimatization in first-time hearing aid users using three different fitting protocols. Aurisn Nasus Larynx 32(4): 345-351.

122. Shanks J, Wilson R, Stelmachowicz P, Gene W, Williams D (2007) Speech-recognition performance after long-term hearing aid use. J Am Acad Audiol 18(4): 292-303.

123. Smeeth L, Fletcher A, Stirling S, Nunes M, Breeze E, et al. (2001) Randomised comparison of three methods of administering a screening questionnaire to elderly people: findings from the MRC trial of the assessment and management of older people in the community. BMJ 323(7326): 1403-1407.

124. Smith SL, Noe CM, Alexander GC (2009) Evaluation of the International Outcome Inventory for Hearing Aids in a Veteran Sample. J Amer Aca dAudiol 20(6): 374-380.

125. Stark P, Hickson L (2004) Outcomes of hearing aid fitting for older people with hearing impairment and their significant others. Int J Audiology 43: 390-398.

126. Swan I, Browing G (1985) Thewispering voice as a screening test for hearing impairment. J R Coll Gen Pract 35: 197.

127. Victor C, Vetter N (1985) Use of community services by the elderly 3 and 12 months after discharge from hospital. Int Rehabil Med 7: 56-59.

128. Stephens D (2002) The International Outcome Inventory for Hearing Aids (IOI-HA) and its relationship to the Client-oriented Scale of Improvement (COSI). Int J Audiol 41(1): 42-47.

129. Takahashi G, Martinez CD, Beamer S, Bridges J, Noffsinger D, et al. (2007) Subjective measures of hearing aid benefit and satisfaction in the NIDCD/VA follow-up study. J Am Acad Audiol 18(4): 323-349.

130. Tomita M, Mann W, Welch T (2001) Use of assistive devices to address hearing impairment by older persons with disabilities. International Journal of Rehabilitation Research 24(4): 279-289.

131. Mann W, Hurren D, Tomita M, Charvat B (1996) Use of assistive devices for bathing by non-institutionalized elderly. The Occupational Therapy Journal of Research 16(4): 261-286.

132. Vestergaard M (2006) Self-report outcome in new hearing-aid users: Longitudinal trends and relationships between subjective measures of benefit and satisfaction. Int J Audiology 45(7): 382-392.

133. EuroQol (1990) EuroQol-a new facility for the measurement of health related quality of life. Health Policy 16: 199-208.

134. Walden T, Walden B (2004) Predicting success with hearing aids in everyday living. J Am Acad Audiol 15(5): 342-352.

135. Williams V, Johnson C, Danhauer J (2009) Hearing Aid Outcomes: Effects of Gender and Experience on Patients' Use and Satisfaction. J Am Acad Audiol 20(7): 422-432.

136.Yueh B, Collins MP, Souza PE, Boyko E, Loovis C, et al. (2010) Long-term effectiveness of screening for hearing loss: the screening for auditory impairment--which hearing assessment test (SAI-WHAT) randomized trial. J Am Geriatr Soc 58(3): 427-434.

137. Kazis L (2000) The Veterans SF-36 Health Status Questionnaire: Development and application in the Veterans Health Administration. Monitor Med Outcomes Trust 5 (1-2): 13-14.

138. Wengraf T (2001) Qualitative Research Interviewing: Biographical Narratives and Semi-Structured Method. London, Sage Publishing.
