# Supplementary material for: A Systematic Review of Studies Measuring and Reporting Hearing Aid Usage in Older Adults since 1999: A Descriptive Summary of Measurement Tools
Source: PLoS One. 2012 Mar 27;7(3):e31831. doi: 10.1371/journal.pone.0031831 (PMC3313982; doi:10.1371/journal.pone.0031831)
Supplement: Table S2 — Quality assessment and grading results. Scoring codes are: 2 (study meets criterion to a high standard); 1 (study partially meets criterion); 0 (study does not meet criterion or relevant information is absent). The grading of the quality of evidence is: High (13–16), Moderate (8–12), and Low (4–7) and Very low (0–3) [19]. Abbreviations: Hearing loss (HL). (DOC) [file pone.0031831.s002.doc]

| **Reference** | **Study design** | **Appropriate outcomes** | **Usage** | **Cross validation** | **Age** | **HL** | **Grade** |
| --- | --- | --- | --- | --- | --- | --- | --- |
| **Arlinger & Billermark, 1999 [48]** | 2 | 2 | 2 | 1 | 2 | 1 | **High** |
| **Banerjee, 2011 [57]** | 0 | 1 | 2 | 0 | 2 | 1 | **Low** |
| **Baumfield & Dillon, 2001 [58]** | 2 | 2 | 0 | 1 | 2 | 0 | **Moderate** |
| **Bertoli et al, 2009 [28]** | 2 | 1 | 2 | 2 | 1 | 0 | **Moderate** |
| **Brannstrom & Wennerstrom, 2010 [42]** | 1 | 1 | 1 | 0 | 1 | 1 | **Low** |
| **Bratt et al, 2007 [27]** | 2 | 2 | 0 | 0 | 2 | 2 | **Moderate** |
| **Chang et al, 2008 [71]** | 1 | 2 | 2 | 0 | 1 | 1 | **Moderate** |
| **Collins et al, 2007 [74]** | 0 | 2 | 2 | 0 | 2 | 2 | **Moderate** |
| **Cook & Hawkins, 2007 [76]** | 0 | 1 | 1 | 0 | 2 | 2 | **Low** |
| **Cox & Alexander, 2002 [77]** | 1 | 1 | 2 | 0 | 2 | 0 | **Low** |
| **Cox et al, 2003 [78]** | 1 | 1 | 1 | 0 | 1 | 2 | **Low** |
| **Cox et al, 2007 [79]** | 1 | 2 | 2 | 0 | 2 | 2 | **Moderate** |
| **Cox et al, 2011 [83]** | 1 | 2 | 2 | 0 | 2 | 1 | **Moderate** |
| **Desjardins & Doherty, 2009 [86]** | 1 | 2 | 2 | 0 | 2 | 2 | **Moderate** |
| **Dillon et al,1999 [14]** | 1 | 2 | 2 | 1 | 1 | 1 | **Moderate** |
| **Gianopoulos et al, 2002 [87]** | 0 | 2 | 2 | 0 | 1 | 2 | **Moderate** |
| **Gnewikow et al, 2009 [39]** | 1 | 2 | 0 | 1 | 0 | 2 | **Low** |
| **Gopinath et al, 2011 [31]** | 1 | 1 | 1 | 0 | 1 | 1 | **Low** |
| **Gussekloo et al, 2003 [43]** | 1 | 1 | 1 | 0 | 1 | 0 | **Low** |
| **Harkins & Tucker, 2007 [93]** | 0 | 1 | 0 | 0 | 1 | 1 | **Very Low** |
| **Hartley et al, 2010 [29]** | 2 | 1 | 2 | 0 | 2 | 1 | **Moderate** |
| **Heuermann et al, 2005 [94]** | 1 | 2 | 2 | 0 | 2 | 0 | **Moderate** |
| **Hickson et al, 1999 [50]** | 0 | 2 | 2 | 1 | 2 | 2 | **Moderate** |
| **Hickson et al, 2010 [96]** | 1 | 2 | 2 | 0 | 2 | 2 | **Moderate** |
| **Hosford-Dunn & Halpern, 2000 [99]** | 1 | 1 | 0 | 1 | 2 | 2 | **Low** |
| **Humes et al, 2001 [11]** | 1 | 2 | 1 | 2 | 2 | 0 | **Moderate** |
| **Humes et al, 2002 [102]** | 0 | 2 | 0 | 1 | 1 | 1 | **Low** |
| **Humes et al, 2004 [47]** | 1 | 2 | 2 | 2 | 1 | 0 | **Moderate** |
| **Humes et al, 2009 [46]** | 2 | 2 | 2 | 2 | 2 | 2 | **High** |
| **Ivory et al, 2009 [40]** | 0 | 1 | 0 | 0 | 0 | 1 | **Very Low** |
| **Jerram & Purdy, 2001 [22]** | 0 | 2 | 2 | 0 | 2 | 2 | **Moderate** |
| **Kam et al, 2011 [107]** | 0 | 1 | 2 | 0 | 2 | 2 | **Moderate** |
| **Keidser et al, 2008 [49]** | 0 | 0 | 1 | 0 | 2 | 1 | **Low** |
| **Kemker & Holmes, 2004 [108]** | 2 | 1 | 2 | 0 | 2 | 2 | **Moderate** |
| **Kricos et al, 2007 [109]** | 1 | 2 | 0 | 1 | 2 | 1 | **Moderate** |
| **Liu et al, 2011 [111]** | 1 | 1 | 2 | 0 | 2 | 2 | **Moderate** |
| **Lockey et al, 2010 [112]** | 0 | 1 | 1 | 0 | 2 | 1 | **Low** |
| **Lupsako & Kautiainen, 2005 [23]** | 1 | 2 | 1 | 0 | 2 | 1 | **Moderate** |
| **Maki-Torkko et al, 2001 [24]** | 1 | 0 | 2 | 2 | 2 | 0 | **Moderate** |
| **Meister et al, 2005 [115]** | 1 | 0 | 1 | 0 | 2 | 1 | **Low** |
| **Munro & Lutman, 2004 [116]** | 0 | 1 | 0 | 0 | 2 | 1 | **Low** |
| **Olusanya, 2004 [117]** | 0 | 1 | 2 | 0 | 2 | 1 | **Low** |
| **Parving & Christensen, 2004 [118]** | 0 | 1 | 2 | 0 | 2 | 2 | **Moderate** |
| **Parving & Sibelle, 2001 [45]** | 1 | 0 | 1 | 0 | 2 | 0 | **Low** |
| **Purdy & Jerram, 2001 [119]** | 0 | 1 | 2 | 0 | 2 | 2 | **Moderate** |
| **Reber & Kompis, 2005 [121]** | 2 | 0 | 2 | 1 | 2 | 1 | **Moderate** |
| **Roup & Noe, 2009 [10]** | 1 | 2 | 2 | 0 | 2 | 0 | **Moderate** |
| **Schneider et al, 2010 [5]** | 1 | 0 | 1 | 0 | 2 | 1 | **Low** |
| **Shanks et al, 2007 [122]** | 1 | 2 | 1 | 0 | 1 | 0 | **Low** |
| **Smeeth et al, 2002 [26]** | 2 | 1 | 1 | 0 | 1 | 0 | **Low** |
| **Smith et al, 2009 [124]** | 1 | 1 | 2 | 0 | 2 | 2 | **Moderate** |
| **Stark & Hickson, 2004 [125]** | 0 | 2 | 2 | 0 | 2 | 1 | **Moderate** |
| **Stephens et al, 2001 [25]** | 1 | 2 | 1 | 0 | 1 | 2 | **Moderate** |
| **Stephens, 2002 [128]** | 0 | 2 | 2 | 0 | 2 | 1 | **Moderate** |
| **Takahashi et al, 2007 [129]** | 1 | 2 | 2 | 1 | 2 | 1 | **Moderate** |
| **Taubman et al, 1999 [16]** | 2 | 2 | 2 | 2 | 2 | 0 | **High** |
| **Tomita et al, 2001 [130]** | 0 | 1 | 1 | 0 | 2 | 2 | **Low** |
| **Uriarte et al, 2005 [9]** | 1 | 2 | 2 | 0 | 2 | 0 | **Moderate** |
| **Vestergaard, 2006 [132]** | 0 | 2 | 0 | 1 | 2 | 2 | **Moderate** |
| **Vuorialho et al, 2006 [44]** | 0 | 0 | 2 | 0 | 1 | 2 | **Low** |
| **Vuorialho et al, 2006 [52]** | 1 | 2 | 2 | 1 | 2 | 2 | **High** |
| **Walden & Walden, 2004 [134]** | 0 | 1 | 2 | 0 | 2 | 2 | **Moderate** |
| **Williams et al, 2009 [135]** | 0 | 1 | 2 | 0 | 2 | 1 | **Low** |
| **Yueh et al, 2010 [136]** | 2 | 2 | 2 | 1 | 2 | 2 | **High** |

**References Table S2**

5. Schneider J, Gopinath B, Karpa M, McMahon C, Rochtchina E, et al. (2010) Hearing loss impacts on the use of community and informal supports. Age Ageing 39: 458-464.

9. Uriarte M, Denzin L, Dunstan A, Sellars J, Hickson L (2005) Measuring hearing aid outcomes using the Satisfaction with Amplification in Daily Life (SADL) questionnaire: Australian data. J Am AcadAudiol 16: 383-402.

10. Roup C, Noe C (2009) Hearing aid outcomes for listeners with high-frequency hearing loss. Am J Audiol, 18: 45-52.

11. Humes L, Garner C, Wilson D, Barlow N (2001) Hearing aid outcome measures following one month of hearing aid use by the elderly. J Speech Hear Res: 443: 469-486.

14. Dillon H, Birtles G, Lovegrove R (1999) Measuring the outcomes of a national rehabilitation program: Normative data for the client oriented scale of improvement (COSI) and the hearing aid user's questionnaire (HAUQ). J Am AcadAudiol 10: 67-79.

16. Taubman L, Palmer C, Durrant J, Pratt S (1999) Accuracy of hearing aid use time as reported by experienced hearing aid wearers. Ear Hear 20: 299-305.

22. Jerram JC, Purdy SC (2001) Technology, expectations, and adjustment to hearing loss: predictors of hearing aid outcome. J Am AcadAudiol 12: 64-79

23. Lupsakko T, Kautiainen H, Sulkava R (2005) The non-use of hearing aids in people aged 75 years and over in the city of Kuopio in Finland. Eur Arch Otorhinolaryngol 262: 165-169.

24. Maki-Torkko E, Sorri M, Laukli E (2001) Objective assessment of hearing aid use. ScandAudiol, 30: 81-82.

25. Stephens D, Lewis P, Davis A, Gianopoulos I, Vetter N (2001) Hearing aid possession in the population: Lessons from a small country. Audiology 40: 104-111.

26. Smeeth L, Fletcher A, Ng ESW, Stirling S, Nunes M, et al. (2002) Reduced hearing, ownership, and use of hearing aids in elderly people in the UK - the MRC Trial of the Assessment and Management of Older People in the Community: a cross-sectional survey. Lancet 359: 1466-1470.

27. Bratt G, Rosenfield M, Williams D (2007) NIDCD/VA hearing aid clinical trial and follow-up: Background. J Am AcadAudiol 18(4): 274-281.

28. Bertoli S, Staehelin K, Zemp E, Schindler C, Bodmer D, et al. (2009) Survey on hearing aid use and satisfaction in Switzerland and their determinants. Int J Audiology 48: 183-195.

29. Hartley D, Rochtchina E, Newall P, Golding M, Mitchell P (2010) Use of Hearing Aids and Assistive Listening Devices in an Older Australian Population. JAm AcadAudiol, 21(10): 642-653.

31. Gopinath B, Mitchell P, Schneider J, Hartley D, Teber E, et al. (2011) Incidence and Predictors of Hearing Aid Use and Ownership among Older Adults with Hearing Loss. Annals of Epidemiology 21(7): 497-506.

39. Gnewikow D, Ricketts T, Bratt G, Mutchler L (2009) Real-world benefit from directional microphone hearing aids. J Rehabil Res Dev 46(5): 603-618.

40. Ivory P, Hendricks B, Van Vliet D, Beyer C, Abrams H (2009) Short-term hearing aid benefit in a large group. Trends Amplif 13(4): 260-280.

42. Brannstrom K J, Wennerstrom I (2010) Hearing aid fitting outcome: clinical application and psychometric properties of a Swedish translation of the international outcome inventory for hearing aids (IOI-HA). J Am Acad Audiol, 21(8): 512-521.

43. Gussekloo J, de Bont LE, von Faber M, Eekhof J, Laat J, et al. (2003) Auditory rehabilitation of older people from the general population--the Leiden 85-plus study. Br J Gen Pract 53: 536-540.

44. Vuorialho A, Sorri M, Nuojua I, Mulhi A (2006) Changes in hearing aid use over the past 20 years. Eu Arch Oto-Rhino-Laryng 263: 355-360.

45. Parving A, Sibelle P (2001) Clinical study of hearing instruments: A cross-sectional longitudinal audit based on consumer experiences. Audiology 40: 43-53.

46. Humes L, Alhstrom J, Bratt G, Peek B (2009) Studies of hearing-aid outcome measures in older adults: A comparison of technologies and an examination of individual differences. Sem Hear 30: 112-128.

47. Humes L, Humes L, Wilson D (2004) A comparison of single-channel linear amplification and tow-channel wide-dynamic-range-compression amplification by means of an independent-group design. Am J Audiol 13: 39-53.

48. Arlinger S, Billermark E (1999) One year follow-up of users of a digital hearing aid. Br J Audiol, 33: 223-232.

49. Keidser G, Hartley D, Carter L (2008) Long-term usage of modern signal processing by listeners with severe or profound hearing loss: a retrospective survey. Am J Audiol 17: 136-146.

50. Hickson L, Timm M, Worrall L, Bishop K (1999) Hearing aid fitting: outcomes for older adults. Aust J Audiology 21(1): 11.

52. Vuorialho A, Karinen P, SorriM (2006) Effect of hearing aids on hearing disability and quality of life in the elderly. Int J Audiol 45(7): 400-405.

57. Banerjee S (2011) Hearing aids in the real world: use of multimemory and volume controls. J Am Acad Audiol 22(6): 359-374.

58. Baumfield A, Dillon H (2001) Factors affecting the use and perceived benefit of ITE and BTE hearing aids. Br J Audiol 35(4): 247-258.

71. Chang W H, Tseng HC, Chao TK, Hsu C, Liu T (2008) Measurement of hearing aid outcome in the elderly: Comparison between young and old elderly. Otolaryngol Head Neck Surg 138(6): 730-734.

74. Collins MP, Souza PE, O'Neill S, Yueh B (2007) Effectiveness of group versus individual hearing aid visits. J Rehabil Res Dev 44(5): 739-749.

76. Cook J, Hawkins D (2007) Outcome measurements for patients receiving hearing aid services. The Laryngoscope 117: 610-613.

77. Cox RM, Alexander GC (2002) The International Outcome Inventory for Hearing Aids (IOI-HA): psychometric properties of the English version. Int J Audiol 41(1): 30-35.

78. Cox RM, Alexander GC, Beyer CM (2003) Norms for the international outcome inventory for hearing aids. J Am Acad Audiol 14(8): 403-413.

79. Cox R, Alexander G, Gray G (2007) Personality, hearing problems, and amplification characteristics: contributions to self-report hearing aid outcomes. Ear Hear 28(2): 141-162.

83. Cox RM, Schwartz KS, Noe CM (2011) Preference for One or Two Hearing Aids among Adult Patients (vol 32, pg 181, 2011). Ear Hear 32(3): 409-409.

86. Desjardins J, Doherty K (2009) Do experienced hearing aid users know how to use their hearing AIDS correctly? Am J Audiol 18(1): 69-76.

87. Gianopoulos I, Stephens D, Davis A (2002) Follow up of people fitted with hearing aids after adult hearing screening: the need for support after fitting. BMJ 325: 471-471.

93. Harkins J, Tucker P (2007) An internet survey of individuals with hearing loss regarding assistive listening devices. Trends Amplif 11(2): 91-100.

94. Heuermann H, Kinkel M, Tchorz J (2005) Comparison of psychometric properties of the International Outcome Inventory for Hearing Aids (IOI-HA) in various studies. Int J Audiol 44(2): 102-109.

96. Hickson L, Clutterbuck S, Khan A (2010) Factors associated with hearing aid fitting outcomes on the IOI-HA. Int J Audiol 49(8): 586-595.

99. Hosford-Dunn H, Halpern J (2000) Clinical application of the satisfaction with amplification in daily life scale in private practice I: statistical, content, and factorial validity. J Am Acad Audiol 11(10): 523-539.

102. Humes L, Wilson D, Barlow N, Barlow N (2002) Changes in hearing-aid benefit following 1 or 2 years of hearing-aid use by older adults. J Speech Hear Res 45(4): 772-782.

107. Kam AC, Tong MC, van Hasselt (2011) Cross-cultural adaptation and validation of the Chinese abbreviated profile of hearing aid benefit. Int J Audiol 50(5): 334-339.

108. Kemker BE, Holmes AE (2004) Analysis of prefitting versus postfitting hearing aid orientation using the Glasgow Hearing Aid Benefit Profile (GHABP). J Am AcadAudiol 15(4): 311-323.

109. Kricos P, Erdman S, Bratt G, Williams D (2007) Psychosocial correlates of hearing aid adjustment. J Am Acad Audiol 18(4): 304-322.

111. Liu H, Zhang H, Liu S, Chen X, Han D, Zhang L (2011) International outcome inventory for hearing aids (IOI-HA): Results from the Chinese version. Int J Audiol 50(10): 673-678.

112. Lockey K, Jennings MB, Shaw L (2010) Exploring hearing aid use in older women through narratives. Int J Audiol: 49(8): 542-549.

115. Meister H, Lausberg I, Kiessling J, von Wedel, H, Walger M (2005) Detecting components of hearing aid fitting using a self-assessment-inventory. Eur Arch Otorhinolaryngol 262(7): 580-586.

116. Munro K, Lutman M (2004) Self-reported outcome in new hearing aid users over a 24-week post-fitting period. Int J Audiol 43(10): 555-562.

117. Olusanya B (2004) Self-reported outcomes of aural rehabilitation in a developing country. Int J Audiol 43(10): 563-571.

118. Parving A, Christensen B (2004) Clinical trial of a low-cost, solar-powered hearing aid. Acta Otolaryngol 124(4): 416-420.

119. Purdy S, Jerram J (2001) Investigation of the profile of hearing aid performance in experienced hearing aid users. Ear Hear 19(6): 473-480.

121. Reber M, Kompis M (2005) Acclimatization in first-time hearing aid users using three different fitting protocols. Aurisn Nasus Larynx 32(4): 345-351.

122. Shanks J, Wilson R, Stelmachowicz P, Gene W, Williams D (2007) Speech-recognition performance after long-term hearing aid use. J Am Acad Audiol 18(4): 292-303.

124. Smith SL, Noe CM, Alexander GC (2009) Evaluation of the International Outcome Inventory for Hearing Aids in a Veteran Sample. J Amer Aca dAudiol 20(6): 374-380.

125. Stark P, Hickson L (2004) Outcomes of hearing aid fitting for older people with hearing impairment and their significant others. Int J Audiology 43: 390-398.

128. Stephens D (2002) The International Outcome Inventory for Hearing Aids (IOI-HA) and its relationship to the Client-oriented Scale of Improvement (COSI). Int J Audiol 41(1): 42-47.

129. Takahashi G, Martinez CD, Beamer S, Bridges J, Noffsinger D, et al. (2007) Subjective measures of hearing aid benefit and satisfaction in the NIDCD/VA follow-up study. J Am Acad Audiol 18(4): 323-349.

130. Tomita M, Mann W, Welch T (2001) Use of assistive devices to address hearing impairment by older persons with disabilities. International Journal of Rehabilitation Research 24(4): 279-289.

132. Vestergaard M (2006) Self-report outcome in new hearing-aid users: Longitudinal trends and relationships between subjective measures of benefit and satisfaction. Int J Audiology 45(7): 382-392.

134. Walden T, Walden B (2004) Predicting success with hearing aids in everyday living. J Am Acad Audiol 15(5): 342-352.

135. Williams V, Johnson C, Danhauer J (2009) Hearing Aid Outcomes: Effects of Gender and Experience on Patients' Use and Satisfaction. J Am Acad Audiol 20(7): 422-432.

136.Yueh B, Collins MP, Souza PE, Boyko E, Loovis C, et al. (2010) Long-term effectiveness of screening for hearing loss: the screening for auditory impairment--which hearing assessment test (SAI-WHAT) randomized trial. J Am Geriatr Soc 58(3): 427-434.
